# Pan-cancer analysis combined with experiments predicts CTHRC1 as a therapeutic target for human cancers

**DOI:** 10.1186/s12935-021-02266-3

**Published:** 2021-10-26

**Authors:** Dazhao Peng, Cheng Wei, Xiaoyang Zhang, Shenghui Li, Hao Liang, Xingyu Zheng, Shulong Jiang, Lei Han

**Affiliations:** 1 Tianjin Neurological Institute, Key Laboratory of Post-Neuroinjury Neuro-Repair and Regeneration in Central Nervous System, Ministry of Education and Tianjin City, Tianjin Medical University General Hospital, 154 Anshan Road, Heping District, Tianjin, 300052 China; 2 Clinical Medical Laboratory Center, Jining First People’s Hospital, Jining Medical University, Jiankang Road, Jining, Shandong 272000 People’s Republic of China; 3 Department of Gynecology and Obstetrics, Tianjin Medical University General Hospital, Tianjin, 300052 China

**Keywords:** CTHRC1, Pan-cancer, Expression, Prognosis, Methylation, Immune

## Abstract

**Background:**

The function of collagen triple helix repeat containing 1 (CTHRC1) as an oncogene has been reported in a growing number of publications. Bioinformatics methods represent a beneficial approach to examine the mechanism and function of the CTHRC1 gene in the disease process of cancers from a pan-cancer perspective.

**Methods:**

In this study, using the online databases UCSC, NCBI, HPA, TIMER2, Oncomine, GEPIA, UALCAN, cBioPortal, COSMIC, MEXPRESS, STRING, CCLE, LinkedOmics, GTEx, TCGA, CGGA, and SangerBox, we focused on the relationship between CTHRC1 and tumorigenesis, progression, methylation, immunity, and prognosis. qPCR was used to detect CTHRC1 expression in glioma tissues and cell lines.

**Results:**

The pan-cancer analysis showed that CTHRC1 was overexpressed in most tumors, and a significant correlation was observed between CTHRC1 expression and the prognosis of patients with cancer. CTHRC1 genetic alterations occur in diverse tumors and are associated with tumor progression. Levels of CTHRC1 promoter methylation were decreased in most cancer tissues compared with normal tissues. In addition, CTHRC1 coordinated the activity of ICP genes through diverse signal transduction pathways, was also associated with immune cell infiltration and the tumor microenvironment, and potentially represented a promising immunotherapy target. We identified CTHRC1-related genes across cancers using the GEPIA2 tool. The single-gene GO analysis of CTHRC1 across cancers showed that it was involved in some signaling pathways and biological processes, such as the Wnt signaling pathway, cell migration, and positive regulation of protein binding. The expression and function of CTHRC1 were also further verified in glioma tissues and cell lines.

**Conclusions:**

CTHRC1 is overexpressed in various cancer types and functions as an important oncogene that may promote tumorigenesis and development through different mechanisms. CTHRC1 may represent an important therapeutic target for human cancers.

**Supplementary Information:**

The online version contains supplementary material available at 10.1186/s12935-021-02266-3.

## Background

Mediated by the transcriptional coactivator β-catenin, the canonical Wnt pathway serves as one of the essential cellular signaling pathways that contributes to controlling embryogenic developmental processes, tissue homeostasis and carcinogenesis [1]. The Wnt/β-catenin canonical pathway and β-catenin-independent noncanonical pathway are activated by Wnt proteins, and the Wnt/calcium (Ca^2+^) pathway and the planar cell polarity pathway have been extensively investigated [2–4]. Highly conserved from lower chordates to mammals, the CTHRC1 protein was identified as a 30 kDa secreted protein and was first verified to be a differentially expressed gene in balloon-injured rat arteries compared with normal rat arteries [5]. Various studies indicate that CTHRC1 regulates tumor progression through CTHRC1/Wnt/β-catenin pathways [6–12]. Mechanistically, by inducing the transcription of downstream target genes (such as cyclin D1, CD44, and c-Myc) and promoting β-catenin nuclear translocation, CTHRC1 ultimately regulates tumor development [8]. Another study showed that the CTHRC1 promoter region is regulated by β-catenin, inducing CTHRC1 transcription [9]. Hence, the interaction network between CTHRC1 and Wnt/β-catenin might accelerate tumor progression. CTHRC1 serves as an essential factor in tumor development and a promising therapeutic target.

CTHRC1 expression at both the mRNA and protein levels is distinctly increased in multiple tumors compared with adjacent normal tissues and has been implicated in tumorigenesis and development, including tumor cell motility, proliferation, invasion, tumor lymph node metastasis, and patient prognosis [13–17]. Additionally, CTHRC1 is involved in inflammatory arthritis, vascular remodeling, bone formation and developmental morphogenesis [18].

In fact, we still lack pan-cancer evidence on the relationship between CTHRC1 and multiple types of tumors. A systematic analysis of CTHRC1 function was performed in multiple convincing online databases to further examine the molecular mechanism by which CTHRC1 affects oncogenesis and the clinical prognosis of patients. In this paper, the role of CTHRC1 in multiple cancers was comprehensively analyzed by examining RNA and protein expression levels, prognosis, genetic alterations, methylation levels, immunology, and relevant cellular pathways. Additionally, we mainly focus on the field of our expertise in neurosurgery. Thus, in combination with the online database analysis and experimental data, we emphatically investigated the expression of CTHRC1 in glioma tissues and cell lines. CTHRC1 is a crucial oncogene that may represent an important target for the effective treatment of cancers. Through this study, we hope to provide new insights into the role of CTHRC1 in the development, treatment and prognosis of human tumors.

## Materials and methods

### Analysis of CTHRC1 gene expression and functions

Based on the UCSC genome browser on human Dec. 2013 (GRCh38/hg38) assembly (http://genome.ucsc.edu/) [19], the genome location information of the CTHRC1 gene was obtained. We also applied the “Gene function” (https://www.ncbi.nlm.nih.gov/gene/) of the National Center for Biotechnology Information (NCBI) database to conduct CTHRC1 mRNA and protein analyses in the “NCBI Reference Sequences (RefSeq)” module.

We logged into the online Human Protein Atlas (HPA) portal (https://www.proteinatlas.org/) and obtained the CTHRC1 gene expression data in different human normal tissues and tumor/nontumor cells by entering the word “CTHRC1” in the “Tissue Atlas”, “Single Cell Type Atlas” and “Cell Atlas” modules. The row data source was TMM normalized. The resulting transcript expression values, denoted Normalized eXpression (NX), were calculated for each gene in every sample. The detailed information is displayed at https://www.proteinatlas.org/about/ assays + annotation. “Low specificity” was defined as “NX ≥ 1 in at least one tissue/region/cell type but not elevated in any tissue/region/cell type.

Then, we logged into the Oncomine database (https://www.oncomine.org/ resource/main.html) [20]and obtained the differences in CTHRC1 gene expression between cancer tissues and normal tissues by entering the word “CTHRC1”. All data were log-transformed, median centered per array, and standard deviation normalized to one per array in this database [20]. We set the thresholds of P-value = 0.001 and fold change = 1.5.

Then, we input CTHRC1 into the “Gene_DE” module of the Tumor Immune Estimation Resource 2.0 (TIMER2) website (http://timer.cistrome.org/) [21] to explore the differences in CTHRC1 expression between diverse tumors of The Cancer Genome Atlas (TCGA) cohorts and their adjacent normal tissues. The row data was normalized using log2 TPM (Transcripts Per Kilobase of exon model per Million mapped reads) transformation. Box plots were constructed to display the distributions of CTHRC1 gene expression levels. The significance of differences in gene expression between tumors and normal tissues was computed using the Wilcoxon test and annotated by the number of stars. White columns indicate that data for normal tissues are not available. Upregulated or downregulated genes in the tumors compared with normal tissues for each cancer type are displayed.

SangerBox (http://SangerBox.com/Tool) is a helpful online portal for TCGA data analysis [22]. We input “CTHRC1” in this web server to investigate the difference in CTHRC1 expression between tumor and normal tissues from datasets in Genotype-Tissue Expression (GTEx) [23] and TCGA databases. The row data was normalized by UCSC database and log2 (TPM + 1) transformation was performed for each expression value. Violin plots display the distributions of gene expression levels. Moreover, the Gene Ontology Biological Process (GO_BP), Gene Ontology Molecular Function (GO_MF) and Gene Ontology Cellular Component (GO_CC) terms of CTHRC1 were explored. The significance of differences in gene expression was determined using a t test, and Pearson’s correlation coefficient was calculated for the Gene Ontology (GO) analysis.

Through the “Pathological Stage Plot” module of Gene Expression Profiling Interactive Analysis 2.0 (GEPIA2) (http://gepia2.cancer-pku.cn/#index) [24], CTHRC1 expression in different pathological stages (stage I-IV) of some TCGA tumors was obtained. Violin plots display the relationship between CTHRC1 expression levels and pathological stages. The TCGA and GTEx gene expression data were re-computed from raw RNA-Seq data by the UCSC Xena project based on a uniform pipeline [24]. The violin plot was constructed using the transformed log2 [TPM + 1].

Finally, we downloaded the CTHRC1 gene expression data for normal tissues, LGG and HGG from the GTEx and TCGA projects. The row data was normalized using log2 TPM transformation. The differences in expression between normal brain tissues and LGG and HGG were analyzed using GraphPad Prism 8.0 software (San Diego, CA, USA) with one-way ANOVA. Meanwhile, we downloaded CTHRC1 mRNA expression data in glioma from the Chinese Glioma Genome Atlas (CGGA) (http://www.cgga.org.cn; dataset ID: mRNAseq_325, mRNAseq_693, and mRNA-array_301) [25]. The row data was merged into a fragments per kilobase transcriptome per million fragments (FPKM) matrix to normalize [25]. Then, we screened the clinical data from patients with World Health Organization (WHO) grades II to IV glioma and used GraphPad Prism 8.0 software to analyze the relationship between the CTHRC1 expression level and tumor grade. One-way ANOVA was used to compare scores between groups. The results were considered statistically significant at a P-value < 0.05.

### Protein expression analysis

We first logged into the online HPA database and obtained the CTHRC1 protein expression data for 44 tissues under physiological conditions by entering the word “CTHRC1”. Indirect ICC-IF labeling of CTHRC1 protein and subcellular localization information was obtained using the “Cell Atlas” module. In addition, images of immunohistochemical staining of histological sections of renal, liver, colorectal, breast and lung cancers and their normal tissues, their prognostic analysis, and the percentage of patients (maximum 12 patients) with high and medium protein expression levels were obtained from the “pathology atlas”.

We applied the “HomoloGene” function of the NCBI database to conduct an analysis of conserved functional domains of the CTHRC1 protein in different species. Additionally, the phylogenetic tree of CTHRC1 in diverse species was obtained using the constraint-based multiple alignment online tool of the NCBI database (https://www.ncbi.nlm.nih.gov/tools/cobalt/).

We logged into the interactive web resource UALCAN portal (http://ualcan.path.uab.edu/analysis-prot.html) [26] and applied the “CPTAC analysis” module to investigate the protein expression level. Within each proteomic profile, the CPTAC database normalized logged expression values to standard deviations from the median [27]. The total CTHRC1 protein expression level was compared between tumors and normal tissues by retrieving “CTHRC1”. The available datasets of six tumors, including ovarian, breast and colon cancers, as well as clear cell RCC, lung adenocarcinoma and UCEC, were selected.

### Survival prognosis analysis

The OS and DFS survival map data for CTHRC1 in all tumor types in TCGA were obtained using the “Survival Map” module of GEPIA2. According to the expression threshold of the cutoff-high (50%) and cutoff-low (50%) values, we obtained the high-expression and low-expression cohorts. Special survival plots with log-rank P-values were obtained using the “Survival Analysis” module of GEPIA2. The result was used in the hypothesis test.

Then, through the interactive operation interface of the Kaplan–Meier plotter (http://kmplot.com/analysis/) [28], we pooled the different Gene Expression Omnibus (GEO) datasets for a series of analyses of OS, DSS, RFS, DMFS, PPS, PFS and FP. The Kaplan–Meier survival plots of breast, liver, lung, ovarian, and gastric cancer cases were generated by entering the word “CTHRC1” in the “mRNA gene chip” and “mRNA RNA-seq” modules. The log-rank P-value, 95% confidence intervals and hazard ratio (HR) were computed.

Additionally, we logged into SangerBox and obtained the COX_OS, COX_DFI and COX_DSS analysis data for different tumors in the “Gene-KM plotter” module by entering “CTHRC1”.

Finally, clinical survival data from patients with glioma were obtained from the CGGA (dataset ID: mRNAseq_693 and mRNAseq_325). We screened clinical data from patients with primary glioma of WHO grade II to IV and used GraphPad Prism 8.0 software to analyze the survival of patients with all WHO grade tumors, WHO grade III-IV tumors and HGG. One-way ANOVA was used to compare the scores between groups. A P-value < 0.05 was considered statistically significant.

### Genetic alteration analysis

We logged into the online cBioPortal database (https://www.cbioportal.org/) [29, 30] to explore the characteristics of CTHRC1 genetic alterations. Then, we selected “TCGA Pan Cancer Atlas Studies” in the “Quick select” section for query. After inputting the word “CTHRC1”, we observed the mutation type, alteration frequency, and CNA data across TCGA tumor datasets in the “Cancer Types Summary” module. The schematic diagram of the protein structure in the “Mutation” module provides information on the overall mutated sites in CTHRC1. After clicking on these sites, we obtained the specific site information in the “Protein Change” section of Excel. Additionally, the survival data for all TCGA tumor samples with or without CTHRC1 genetic alterations are displayed in the “Comparison/Survival” module. A log-rank P-value < 0.05 was considered significant.

The catalogue of Somatic Mutations in Cancer (COSMIC) (http://www.sanger.ac.uk/cosmic/) [31] is the largest public resource for information on somatically acquired mutations in human cancers. We obtained the mutation distribution of CTHRC1 in module of “Mutational Signatures” and the mutation site of CTHRC1 protein via function of COSMIC-3D.

After logging into Assistant for Clinical Bioinformation (https://www.aclbi.com/static/index.html#/), we downloaded the clinical data, transcriptome data, and CTHRC1 genetic mutation data from TCGA database. We also used the “maftools” package in R software (R Foundation for Statistical Computing, Vienna, Austria, RRID:SCR_003302) to download and visualize the somatic mutations of patients with UCEC across TCGA databases. Genes with a higher mutation frequency in patients with UCEC are displayed in a horizontal histogram.

### DNA methylation analysis

Using the “TCGA gene analysis” function of the UALCAN portal, we explored the difference in CTHRC1 DNA promotor methylation levels between tumor and normal tissues. The database used TPM to normalize the methylation expression value of row data from TCGA [26]. The CTHRC1 DNA promotor methylation levels in thirteen tumors were analyzed.

Then, CTHRC1 mRNA expression (RNA-seq and microarray) and DNA methylation (RRBS) data in glioma were obtained from the Cancer Cell Line Encyclopedia (CCLE) portal (https://portals.broadinstitute.org/ccle/) [32]. The relationship between CTHRC1 mRNA expression and the DNA methylation level was analyzed using GraphPad Prism 8.0 software. Pearson’s correlation coefficient was calculated to assess the association of CTHRC1 mRNA expression and DNA methylation levels.

Finally, we logged into the MEXPRESS website (https://mexpress.be/) [33]and input “CTHRC1” to investigate the DNA methylation level of CTHRC1 in LGG and GBM. The RNA-seq row data was log-transformed before being used to draw the plots in MEXPRESS website [33]. The Benjamini-Hochberg-adjusted P-value and Pearson’s correlation coefficient (R) value were obtained. The promoter region probes (e.g., cg07529715, etc.) were highlighted.

### Analysis of immune cell infiltration

We logged on to the online Tumor Immune Estimation Resource (TIMER) portal (https://cistrome.shinyapps.io/timer/) to analyze the abundance of TIICs from gene expression profiles obtained from TCGA cancer cases [34, 35]. Using the “gene” module in TIMER, we analyzed the association of CTHRC1 expression with the abundance of infiltrating immune cells, including CD8 + T cells, CD4 + T cells, B cells, neutrophils, macrophages, and dendritic cells. We then used the “Immune-Gene” module of TIMER2 to explore the association between CTHRC1 expression and CD8 + T cell infiltration. The “Purity Adjustment” option was selected to perform Spearman’s correlation analysis. The results for different cancer types are shown in a heatmap with numbers. A scatter plot that presents the relationship between the infiltrate estimation value and gene expression in different cancers was obtained by clicking the cells on the heatmap. We obtained the P-values and partial correlation (cor) values.

We then logged into the SangerBox website with the query “CTHRC1” to investigate the relationship between CTHRC1 expression and MSI, ESTIMATE, various immune cells, and ICP in different tumors from TCGA cohorts. Spearman’s rank correlation test was performed, and the P-value and partial correlation (cor) value were generated.

### CTHRC1-related gene enrichment analysis

By searching the STRING website (https://string-db.org/) [36], we queried “CTHRC1” in the protein name module and “*Homo sapiens*” in the organism module. We then set the following main parameters: meaning of network edges (“evidence”), active interaction sources (“experiments” and “database”), minimum required interaction score [“low confidence (0.150)”], and maximum number of interactors to show (“no more than 50 interactors” in the 1st shell). Finally, we observed the available CTHRC1-binding proteins.

The top 10 CTHRC1-correlated targeting genes in TCGA tumor and normal tissues were obtained using the “Similar Gene Detection” function of GEPIA2. Subsequently, a Pearson correlation analysis of CTHRC1 and these 10 genes was performed by applying the “correlation analysis” module of GEPIA2. The correlation coefficient (R) and the P-value were generated. The log2 TPM was used to generate the scatter plot. Moreover, by applying the “Gene_Corr” function of TIMER2, we obtained a heatmap of these top 10 targeting genes.

The LinkedOmics database (http://www.linkedomics.org/login.php) [37] was used to analyze genes coexpressed with CTHRC1, Kyoto Encyclopedia of Genes and Genomes (KEGG) pathways and GO_BP terms in glioma (GBM/LGG). We set the following main parameters: select search dataset (“RNA-seq data type, HiSeq RNA platform”), select search dataset attribute (“CTHRC1”), select target dataset (“RNA-seq data type, HiSeq RNA platform”), and select statistical method (“Pearson’s correlation test”). CTHRC1 coexpression is presented in heat maps. The KEGG pathways and GO_BP terms were examined using the gene set enrichment analysis (GSEA) function module. The rank criterion was an FDR < 0.05, and 1000 simulations were performed.

### Clinical tissue samples and cell lines

All clinical samples were collected from patients with primary glioma who underwent surgery at the First Affiliated Hospital of Zhengzhou University. A microscopic examination of each sample confirmed ≥ 80% tumor cells. These samples were quickly stored in liquid nitrogen. According to the WHO criteria, the neuropathologist verified the pathological grade of each tissue sample. These glioma specimens included 19 grade II gliomas, 9 grade III gliomas, and 15 grade IV gliomas. Detailed information on the glioma samples is provided in Additional file 12: Table S1. This study was approved by the institutional review boards of the hospitals, and written informed consent was obtained from all patients.

Human astrocytes and human glioma cell lines (U87, LN229, U251, A172, and T98G) were obtained from ATCC (American Type Culture Collection, Manassas, VA, USA). Through the ATCC cell line authentication service, cell lines were tested, and mycoplasma was also routinely tested. Human astrocytes were grown in astrocyte medium (AM) (Cat. #1801, ScienCell, USA) supplemented with 10 mL of fetal bovine serum (FBS) (Cat. #0010, ScienCell, USA), 5 mL of Astrocyte Growth Supplement (AGS) (Cat. #1852, ScienCell, USA) and 5 mL of penicillin/streptomycin solution (P/S) (Cat. #0503, ScienCell, USA). Human glioma cell lines were maintained in Dulbecco’s modified Eagle’s medium (DMEM) supplemented with 10% FBS (Thermo Fisher Scientific, USA). All cell lines were grown in a humidified incubator with 5% CO_2_ at 37 °C.

### qPCR experiments

Total RNA was extracted from glioma tissues and cell lines using TRIzol reagent (Thermo Fisher Scientific, USA). Using a reverse transcription kit (Promega, USA), the RNA (3 μg) was reverse transcribed into cDNAs with a reverse transcription system of 20 μL. Quantitative polymerase chain reaction (qPCR) was conducted using 2 × SYBR Green qPCR Master Mix (Low ROX) (Cat #: B21702, Bimake, USA). The reaction mixture volume was 20 μL, including 10 μL of 2 × SYBR Green qPCR Master Mix (Low ROX), 6 μL of nuclease-free water, 0.2 μM of each primer and 2 μL of cDNA products. The PCR cycling conditions were as follows: 95 °C for 3 min, 95 °C for 15 s, and 60 °C for 40 s for 1 cycle and 95 °C for 15 s, 60 °C for 1 min, and 95 °C for 1 s for 40 cycles followed by the melting curve stage. Relative gene expression was obtained using the 2^−ΔΔCT^ method. A t test was used for statistical analyses, and P < 0.05 was considered statistically significant.

The following primer sequences were used:

GAPDH

Forward: 5′-GGTGGTCTCCTCTGACTTCAACA-3′,

Reverse: 5′-GTTGCTGTAGCCAAATTCGTTGT-3’,

CTHRC1

Forward: 5′-GGACCAAGGAAGCCCTGAAAT-3’, and.

Reverse: 5’-AGCAACATCCACTAATCCAGCA-3’.

The relative standard curve method was used to analyze the data, which were normalized to GAPDH.

### Statistical analysis

The HR and P-value were used to evaluate the significance of differences in survival. Pearson’s correlation coefficient and statistical significance were used to assess the associations of gene expression, and the absolute value was used to determine the strength of the correlation. The results were regarded as statistically significant at *P < 0.05, **P < 0.01 and ***P < 0.001.

## Results

### Analysis of CTHRC1 gene expression and function in multiple cancers

**Fig. 1:**
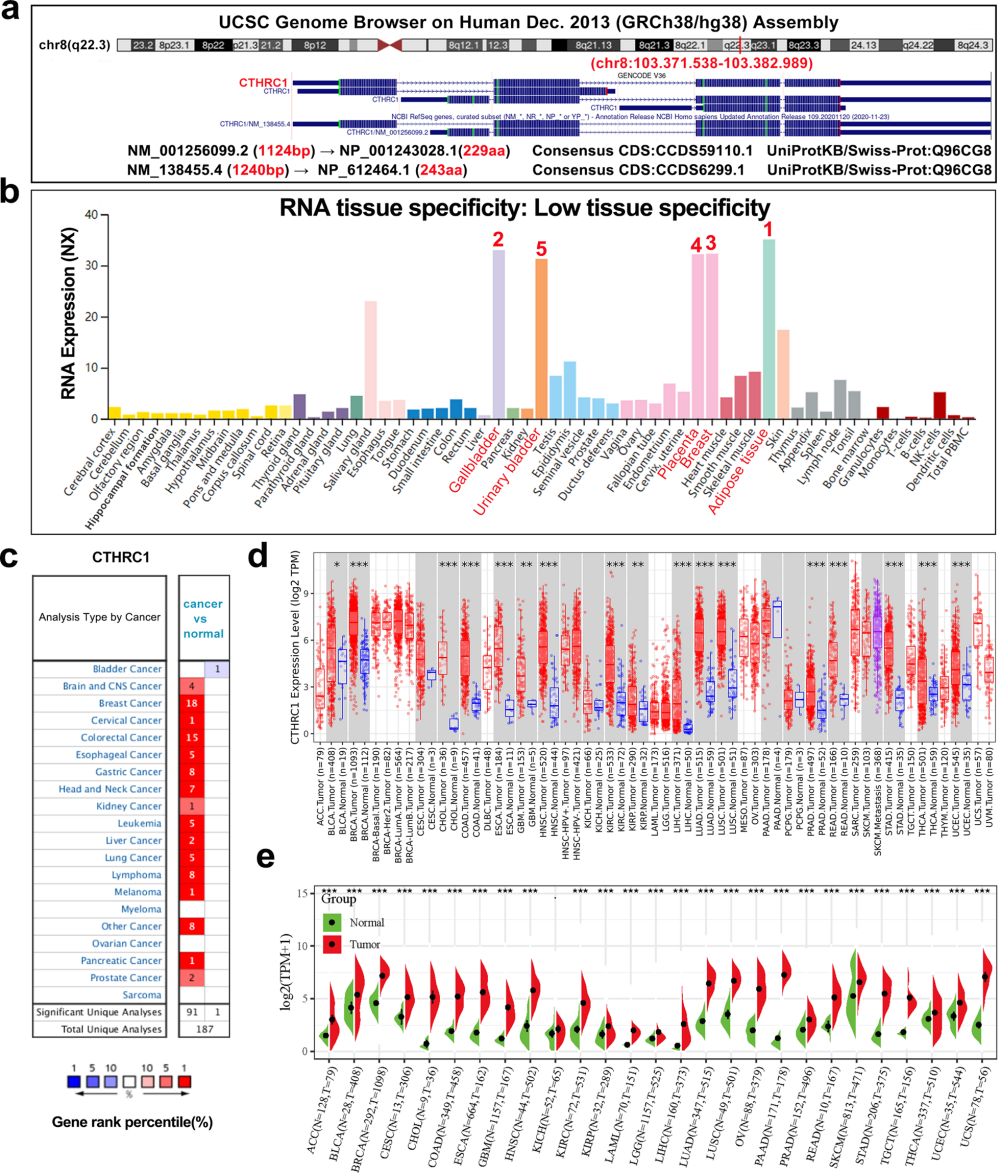
CTHRC1 characteristics and its expression levels in normal tissues and cancers. **a** Genomic location of human CTHRC1 gene; **b** CTHRC1 consensus Normalized eXpression (NX) levels for 55 normal tissue types and 6 blood cell types, created by combining the data from the three transcriptomics datasets (HPA, GTEx and FANTOM5); **c** Increased or decreased CTHRC1 mRNA in data sets of different cancers compared with normal tissues in the Oncomine database; **d** Human CTHRC1 mRNA expression levels in different tumor types from TCGA database were determined by TIMER; **e** The differences in expression levels of CTHRC1 mRNA in different tumors and normal tissues from TCGA and GTEx database were determined by SangerBox; (*P < 0.05, ** P < 0.01, *** P < 0.001)

The analysis process is shown in Additional file 1: Fig. S1. We attempted to investigate the role of CTHRC1 in human cancers. The CTHRC1 gene has two mRNA transcript variants (NM_001256099.2 and NM_138455.4) and two protein isoforms (collagen triple helix repeat-containing protein 1 isoform 2, NP_001243028.1 and collagen triple helix repeat-containing protein 1 isoform 1 precursor, NP_612464.1) (Fig. 1a).

We first performed an analysis of the pattern of CTHRC1 expression in various normal tissues and tumor/nontumor cells. As shown in Fig. 1b, CTHRC1 was expressed at high levels in adipose tissue, gallbladder, breast, placenta and urinary bladder (NX > 30). Although CTHRC1 expression is low in most normal tissues, it is detectable (NX > 1), indicating that the CTHRC1 mRNA has low tissue specificity. In addition, CTHRC1 single-cell type specificity is shown in Additional file 2: Fig. S2a. The CTHRC1 expression level was significantly higher in fibroblasts (NX > 150), melanocytes and endothelial cells. Exocrine glandular cells, blood and immune cells and some other cell types do not express CTHRC1. However, among tumor/nontumor cell lines, the CTHRC1 expression levels in a muscle cell line (HSkMC), skin cells (WM-115, NX > 70), and mesenchymal cells (ASC TERT1, fHDF/TERT166, and HHSteC, NX < 60) are shown in Additional file 2: Fig. S2b. These results revealed the different expression patterns of the CTHRC1 mRNA in normal tissues and tumor/nontumor cell lines, which contributed to our understanding of the specificity of CTHRC1 expression.

We analyzed the differences of CTHRC1 mRNA expression levels in different cancer and normal tissues using the Oncomine database. The CTHRC1 expression level was enhanced in brain and CNS cancer, breast cancer, cervical cancer, colorectal cancer, esophageal cancer, gastric cancer, head and neck cancer, kidney cancer, liver cancer, lung cancer, ovarian cancer, pancreatic cancer, prostate cancer and leukemia, lymphoma and melanoma tumors compared to normal tissues (Fig. 1c). The detailed results of CTHRC1 expression in various tumors are summarized in Additional file 12: Table S2.

To further explore CTHRC1 expression in human cancers, we examined CTHRC1 expression using the TIMER2 and SangerBox online databases. The different expression statuses of CTHRC1 across various cancer types in TCGA are displayed in Fig. 1d. CTHRC1 expression levels were increased in various tumor tissues, including BLCA, BRCA, CHOL, COAD, ESCA, GBM, HNSC, KIRC, KIRP, LIHC, LUAD, LUSC, PRAD, READ, STAD, THCA and UCEC. Compared with their adjacent normal tissues, a significant difference in CTHRC1 expression was observed in ACC, BLCA, BRCA, CESC, CHOL, COAD, ESCA, GBM, HNSC, KIRC, KIRP, LAML, LGG, LIHC, LUAD, LUSC, OV, PAAD, PRAD, READ, SKCM, STAD, TGCT, THCA, UCEC and UCS (Fig. 1e). However, the expression of CTHRC1 in KICH, MESO, PCPG, THYM, and UVM did not differ significantly in either database.

Additionally, the relationship between the CTHRC1 expression level and various cancer pathological stages was analyzed using GEPIA2. Overall, CTHRC1 expression has been positively implicated in the pathological stages of various cancers, including ACC, BLCA, ESCA, KICH, KIRC, KIRP, LUSC, PAAD, STAD and THCA (Additional file 2: Fig. S2c, all P < 0.05). Then, we downloaded CTHRC1 expression data for glioma from GTEx, TCGA and CGGA datasets. Analyses of both the CGGA and TCGA datasets indicated that the CTHRC1 mRNA was consistently upregulated with increasing grade in glioma samples (Additional file 3: Fig. S3a, b).

To further explore the function of CTHRC1, we performed a single-gene GO analysis of CTHRC1 in SangerBox. The results indicated that CTHRC1 is involved in biological processes, such as cell migration, positive regulation of protein binding, positive regulation of osteoblast proliferation, and negative regulation of the canonical Wnt signaling pathway. Functioning as a cellular component, CTHRC1 mRNA was located in the cytoplasm, and the CTHRC1 protein was part of the collagen trimer or collagen-containing extracellular matrix and secreted to extracellular region. In terms of molecular functions, CTHRC1 was involved in frizzled binding, extracellular matrix structural constituent, Wnt-protein binding (Additional file 3: Fig. S3c and Additional file 12: Table S3).

Collectively, these results indicate that CTHRC1 may be a key oncogene in multiple human tumors and closely related to the tumor stage. Functionally, CTHRC1 was involved in the canonical Wnt signaling pathway and played an important pathophysiological role in tumors.

### Analysis of CTHRC1 protein expression in human cancers

**Fig. 2:**
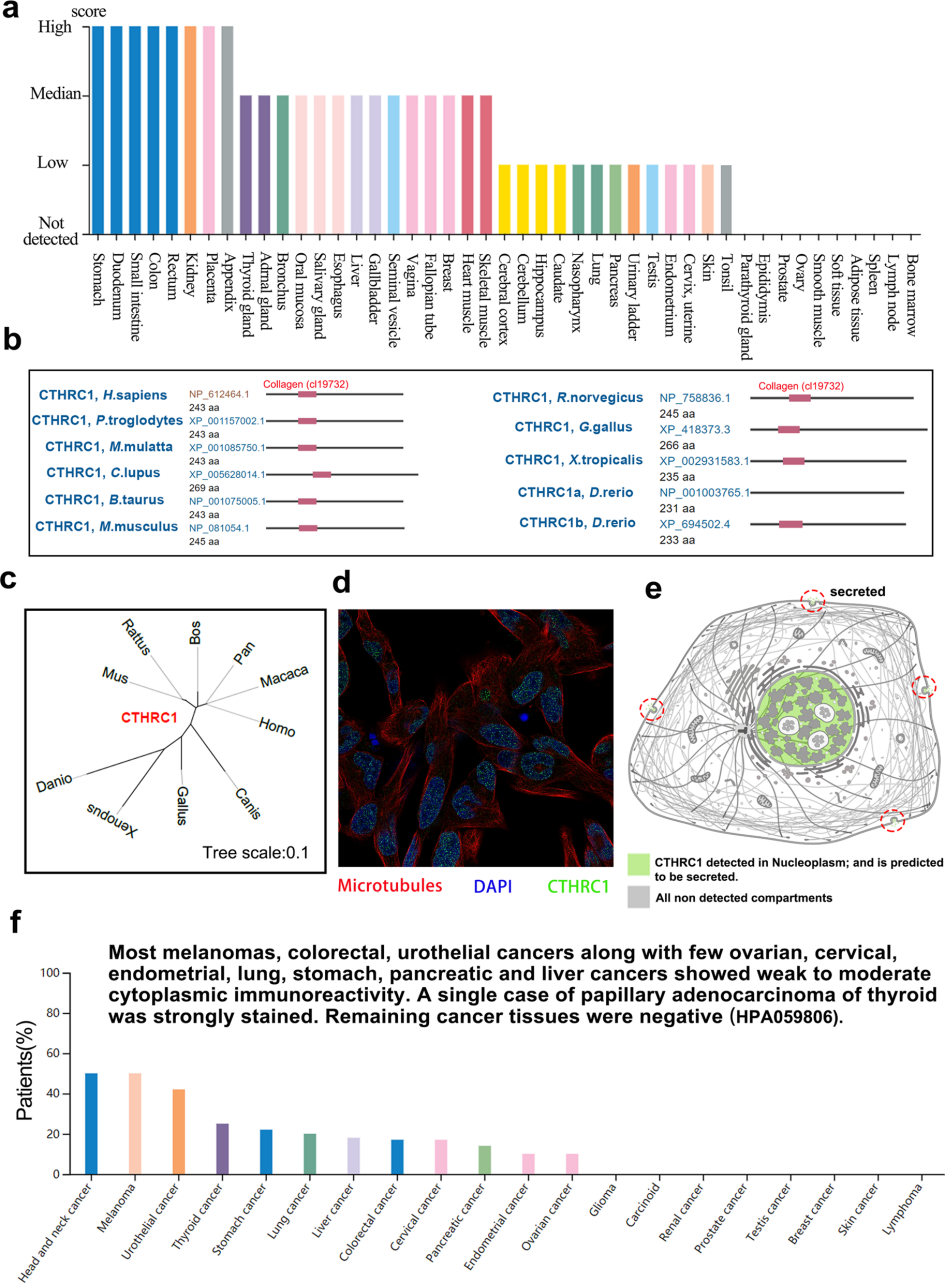
CTHRC1 protein expression, conservation and location. **a** CTHRC1 protein expression data in 44 normal tissues; **b** Conservation of CTHRC1 protein among different species; **c** The phylogenetic tree of CTHRC1 in different species; **d** CTHRC1 protein is sited in the nucleus of RH-30 cell, DAPI for the nucleus in blue, the CTHRC1 protein staining is shown in green; **e** CTHRC1 protein can be secreted (red circle); **f** The percentage of cancer patients (maximum 12 patients) with high and medium protein expression level

We have shown that CTHRC1 mRNA is abnormally regulated in various cancers, and thus we continued to explore the role of the CTHRC1 protein in cancers. In the HPA database, 44 normal tissues were examined for CTHRC1 protein expression. The CTHRC1 protein was expressed at a high level in the stomach, duodenum, small intestine, colon, rectum, kidney, placenta and appendix but was not detected in the parathyroid gland, epididymis, prostate, ovary, smooth muscle, soft tissue, adipose tissue, spleen, lymph node or bone marrow (Fig. 2a). Then, we analyzed the conservation of the CTHRC1 protein in the NCBI database. The collagen (cl19732) domain of the CTHRC1 protein is conserved among multiple species (Fig. 2b). The evolutionary relationship of the CTHRC1 protein across different species is presented in a phylogenetic tree (Fig. 2c). Additionally, the CTHRC1 protein was localized in the nucleus of RH-30 (metastatic rhabdomyosarcoma cell line) cells and secreted extracellularly (Fig. 2d-e). The IHC results showed higher expression of the CTHRC1 protein in tissues of renal, liver, colorectal, lung, and breast cancers than in normal tissues, and higher CTHRC1 expression in liver and renal cancer was distinctly associated with the patient prognosis (P < 0.001) (Additional file 4: Fig. S4a). In patient samples, weak to moderate cytoplasmic immunoreactivity was observed in most melanomas and colorectal and urothelial cancers, as well as a few ovarian, cervical, endometrial, lung, stomach, pancreatic and liver cancers (Fig. 2f). A single case of papillary adenocarcinoma of the thyroid was intensely stained. Other tumor tissues were negatively stained. Finally, the analysis of the CPTAC dataset showed that the expression levels of the total CTHRC1 protein were enhanced in primary breast cancer, ovarian cancer, colon cancer, clear cell RCC, UCEC and LUAD compared with normal tissues (Additional file 4: Fig. S4b). Therefore, these results at the protein level suggest that CTHRC1 is associated with cancer.

### Analysis of the relationship between CTHRC1 expression and prognosis

**Fig. 3:**
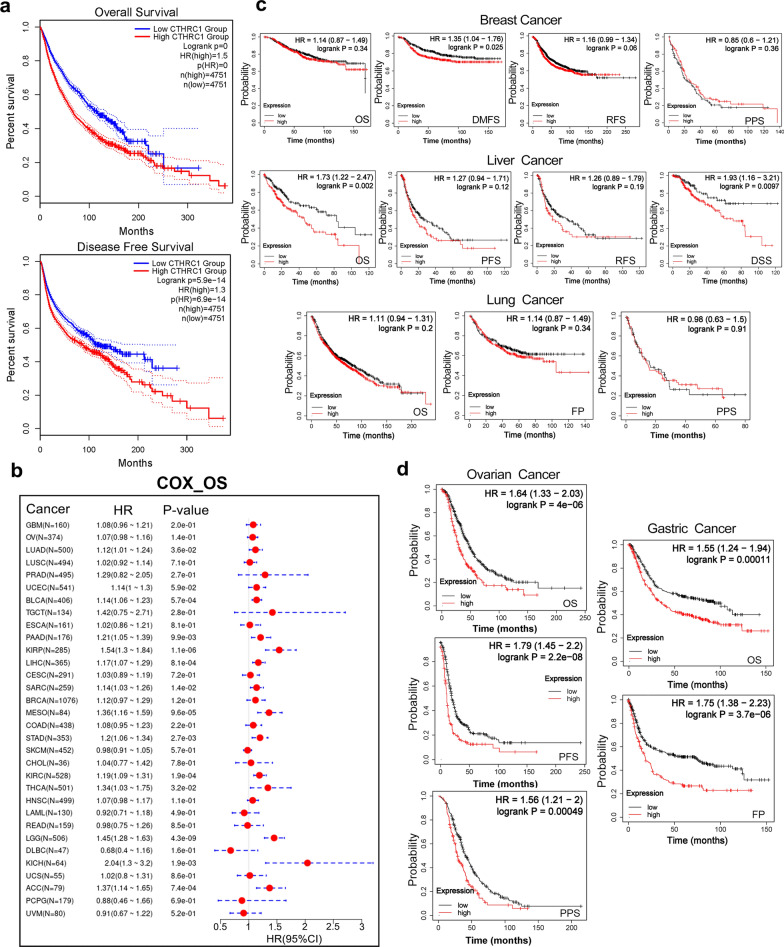
Correlation between CTHRC1 mRNA expression and prognosis of cancers. **a** We used the GEPIA2 tool to perform overall survival and disease-free survival analyses of pan-cancer in TCGA by CTHRC1 mRNA expression; **b** The relationships between CTHRC1 expression and OS prognosis of different cancers in “Gene-KM plotter” module of SangerBox. The Kaplan–Meier plotter was performed survival analyses, including OS, DMFS, RFS, PPS, FP DSS, via the expression level of the CTHRC1 mRNA in breast cancer, liver cancer and lung cancer cases **c**, and OS, PFS, PPS, FP, via the expression level of the CTHRC1 mRNA in ovarian cancer and gastric cancer (**d**)

We performed OS and DFS analyses of pan-cancer cohorts as well as the prognosis of patients with different cancers using GEPIA2. High CTHRC1 expression levels were associated with shorter OS and DFS across cancers (Fig. 3a). Moreover, CTHRC1 overexpression was linked to poor OS prognosis for patients with ACC (P = 0.00039), BLCA (P = 0.045), COAD (P = 0.018), KIRP (P = 0.029), LGG (P = 0.015), LIHC (P = 0.0094), MESO (P = 0.025) and SARC (P = 0.0059) within TCGA cohorts (Additional file 5: Fig. S5a). However, low CTHRC1 gene expression predicted a poor OS prognosis for patients with SKCM (P = 0.041). High CTHRC1 expression was negatively correlated with DFS of patients with ACC (P < 0.0001), COAD (P = 0.015), KIRC (P < 0.0001), KIRP (P = 0.027), and PRAD (P = 0.0064) (Additional file 5: Fig. S5b). The Cox analysis in SangerBox database revealed that CTHRC1 expression was a factor influencing OS, DSS and DFI in patients with various tumors (Fig. 3b, Additional file 6: Fig. S6a, b).

In addition, using the Kaplan–Meier plotter portal, we found that CTHRC1 expression was related to the DMFS (P = 0.025), but was not associated with PPS (P = 0.36), RFS (P = 0.06) and OS (P = 0.34) of patients with breast cancer (Fig. 3c). High CTHRC1 expression was associated with shorter OS (P = 0.002) and DSS (P = 0.0097), but was not associated with RFS (P = 0.19) or PFS (P = 0.12) for patients with liver cancer (Fig. 3c). However, CTHRC1 expression was not associated with OS (P = 0.2), PPS (P = 0.91), FP (P = 0.34) of lung cancer patients (Fig. 3c). Similarly, high CTHRC1 expression was implicated in a shorter OS (P < 0.0001), PFS (P < 0.0001) and PPS (P = 0.00049) of patients with ovarian cancer (Fig. 3d). High CTHRC1 expression signified poor OS (P = 0.00011) and FP (P < 0.0001) prognoses for patients with gastric cancer (Fig. 3d).

In particular, we focused on the association between CTHRC1 expression and the glioma prognosis. Based on the CGGA cohort, the correlation between the CTHRC1 mRNA expression level and overall survival of patients with different grades of glioma was further analyzed using Kaplan–Meier survival curves and the log-rank method. The analysis of CGGA mRNAseq_325 data showed that patients with glioma presenting higher CTHRC1 expression levels had a worse survival probability than those with lower CTHRC1 expression levels. Moreover, CTHCRC1 expression was inversely associated with overall survival in patients with HGG (Additional file 6: Fig. S6c). We performed the same analysis on the CGGA mRNAseq_693 data and obtained similar results (Additional file 6: Fig. S6d). Overall, these results confirmed that a higher CTHRC1 expression level predicts a poorer prognosis in patients with various cancers.

### Analysis of CTHRC1 genetic alterations in different cancers

**Fig. 4:**
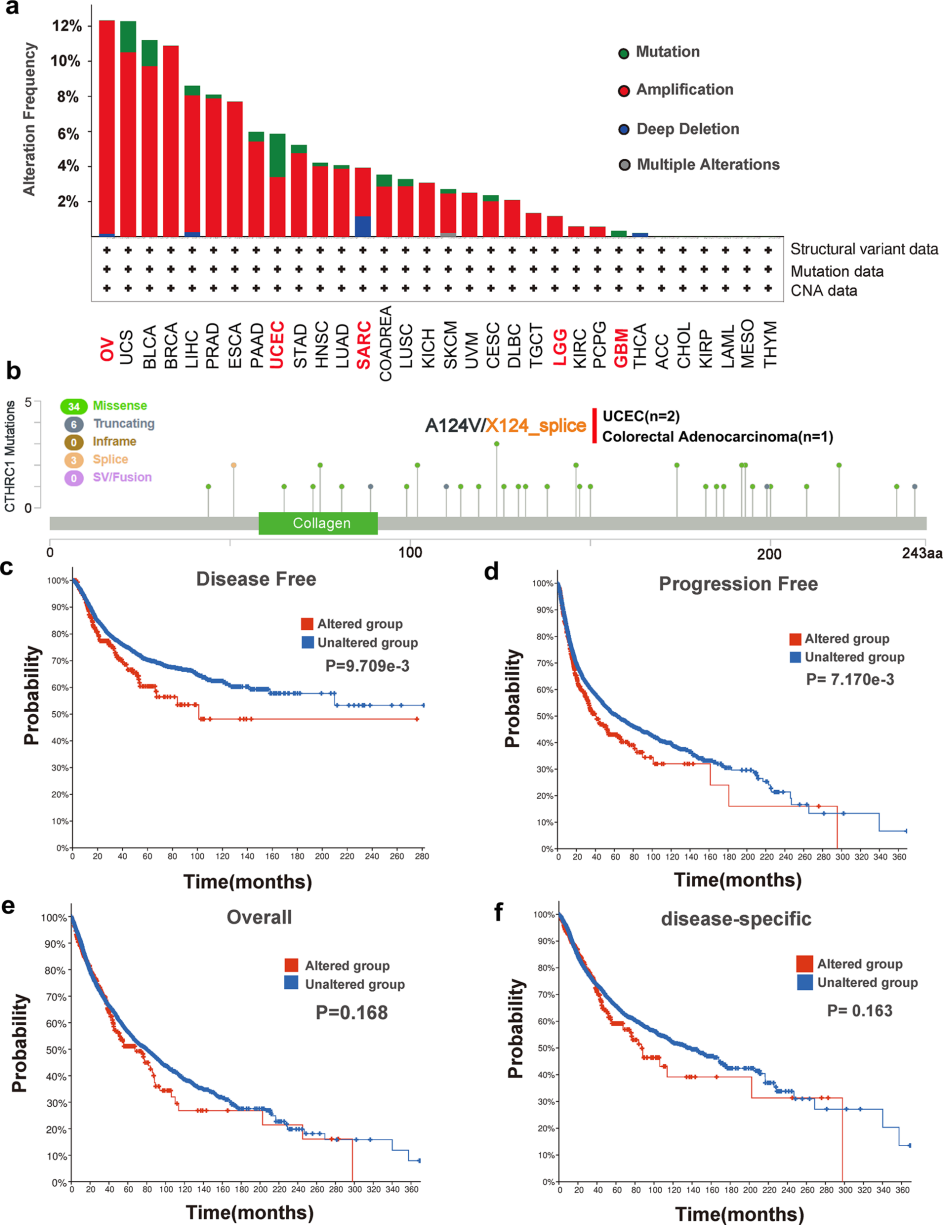
Mutation feature of CTHRC1 in different tumors. Mutation features of CTHRC1 for tumors was analyzed using the cBioPortal tool. The alteration frequency with mutation type **a** and mutation site **b** are displayed. The potential correlation between mutation status and disease-specific was also analyzed **c**, progression-free **d**, overall **e** and disease-free **f** survival of all TCGA tumors using the cBioPortal tool

Through the online database cBioPortal, CTHRC1 genetic alteration information was investigated in various tumor samples from TCGA datasets (Fig. 4a). Genetic alterations in CTHRC1 were dominated by “amplification” types, which were observed in almost all TCGA cancer cases, and the “mutation” type was the second most common. The highest frequency CTHRC1 alteration (> 12%) was observed in patients with ovarian serous cystadenocarcinoma, with “amplification” as the primary type. With an alteration frequency of ~ 2%, the “mutation” type predominated in the UCEC samples. The “deep deletion” type of cancer was rare and only detected in SARC, which showed an alteration frequency of ~ 1%. In LGG and GBM, amplification and mutation were the main alterations in CTHRC1, but were only present at approximately 1%.

Sequentially, we explored special information on CTHRC1 genetic alterations across different tumors. The results revealed that missense mutation of CTHRC1 was identified as the primary type of genetic alteration (Fig. 4b, Additional file 7: Fig. S7a and Additional file 13), and was mainly observed at position of 75 and 124 (Additional file 7: Fig. S7b). The primary SNV class type was G > A (Additional file 7: Fig. S7c). The A124V/X124-splice alteration was detected in 2 UCEC cases and 1 colorectal adenocarcinoma case (Fig. 4b). In addition, a potential correlation between CTHRC1 genetic alterations and the clinical survival prognosis of patients in pan-cancer samples was also detected. A better prognosis of disease-free (P = 9.709e-3) and progression-free (P = 7.170e-3) survival was observed for patients with tumors lacking CTHRC1 alterations, but not overall (P = 0.168) and disease-specific (P = 0.163) survival, compared to patients with CTHRC1 alterations (Fig. 4c-f). Based on this result, CTHRC1 alterations may be involved in cancer progression.

Since the highest mutation frequency was observed for CTHRC1 in UCEC, we focused on analyzing mutations in this tumor. A lollipop plot displays the mutation distribution in CTHRC1 protein (Additional file 8: Fig. S8a). The CTHRC1 gene had a 2% mutation frequency in patients with UCEC (Additional file 8: Fig. S8b). Missense mutation types were predominant in patients with UCEC (Additional file 8: Fig. S8c and d). The primary SNV class type was C > T (Additional file 8: Fig. S8e). Additional file 8: Fig. S8f shows the number of mutations per sample. SNP was the most common variant type compared with INS or DEL (Additional file 8: Fig. S8g). The top ten mutated genes are shown in Additional file 8: Fig. S8h, including TTN, MUC16, PTEN, CSMD3, PIK3CA, ARIDIA, KMT2D, TP53, PIK3RI and CTCF. CTHRC1 genetic alterations in other cancer types require further exploration.

### CTHRC1 DNA methylation analysis

**Fig. 5:**
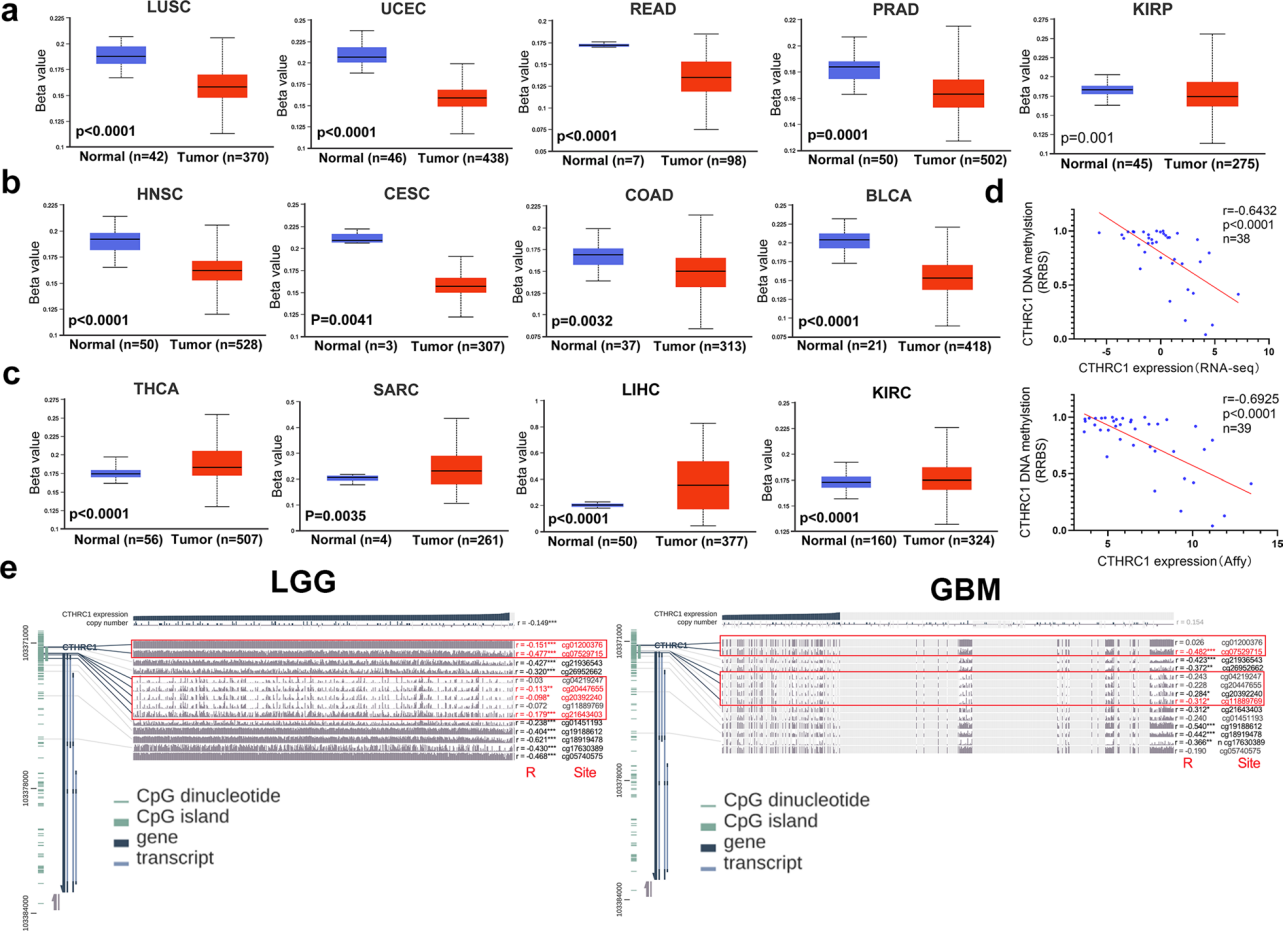
Methylation level of CTHRC1 DNA in different tumors and its association with gene expression. **a**–**c** Promoter methylation levels of CTHRC1 in different cancer types compared to normal adjacent tissues. The Beta value indicates level of DNA methylation ranging from 0 (unmethylated) to 1 (fully methylated). Different beta value cut-off has been considered to indicate hyper-methylation [Beta value: 0.7—0.5] or hypo-methylation [Beta-value: 0.3—0.25]. **d** The relationship between the CTHRC1 mRNA expression (RNA-seq or Affy) and DNA methylation in glioma in CCLE database. **e** We used the MEXPRESS approach to analyze the DNA methylation level of CTHRC1 with multiple probes. The promoter region probes are highlighted by red rectangle. The beta value of methylation, the Benjamini-Hochberg-adjusted P-value and the Pearson correlation coefficients (R) are displayed

Aberrant methylation is linked to oncogenesis, and differences in the methylation patterns distinguish between tumors and benign tissues [38–40]. Methylation may be a promoter or an inhibitor of tumor formation. Therefore, we analyzed differences in the level of CTHRC1 promoter methylation between tumors and adjacent normal tissues using the UALCAN. Levels of CTHRC1 promoter methylation in LUSC, UCEC, READ, PRAD, KIRP, HNSC, CESC, COAD and BLCA were reduced compared with those in their adjacent normal tissues (Fig. 5a, b). In contrast, the level of CTHRC1 promoter methylation in THCA, SARC, LIHC and KIRC was higher than that in their adjacent normal tissues (Fig. 5c). The relationship between the CTHRC1 methylation level and prognosis also requires further exploration.

CCLE data of glioma analysis indicated the CTHRC1 mRNA expression level was negatively linked to the CTHRC1 DNA methylation level in both CTHRC1 mRNA expression (Affy and RNA-seq) datasets (Fig. 5d). Therefore, we subsequently investigated the potential correlation between CTHRC1 DNA methylation and the pathogenesis in glioma from TCGA cohort via the MEXPRESS approach (Fig. 5e). Overall, DNA methylation levels were negatively associated with the glioma grade. Importantly, for the cg07529715 probe of the promoter region, the methylation level decreased with increasing glioma grade. Moreover, CTHRC1 DNA methylation at numerous probes in the nonpromoter region, such as cg17630389 (LGG, P < 0.0001, R = -0.430; GBM, P = 0.0029, R = -0.366), was distinctly negatively correlated with gene expression.

### CTHRC1 expression is related to immunity

Tumor-infiltrating immune cells are the primary components of the tumor microenvironment and exert important effects on the initiation, progression or metastasis of cancer [41, 42]. Therefore, we evaluated the associations of CTHRC1 expression with the levels of immune cell infiltration in 39 cancer cases.

**Fig. 6:**
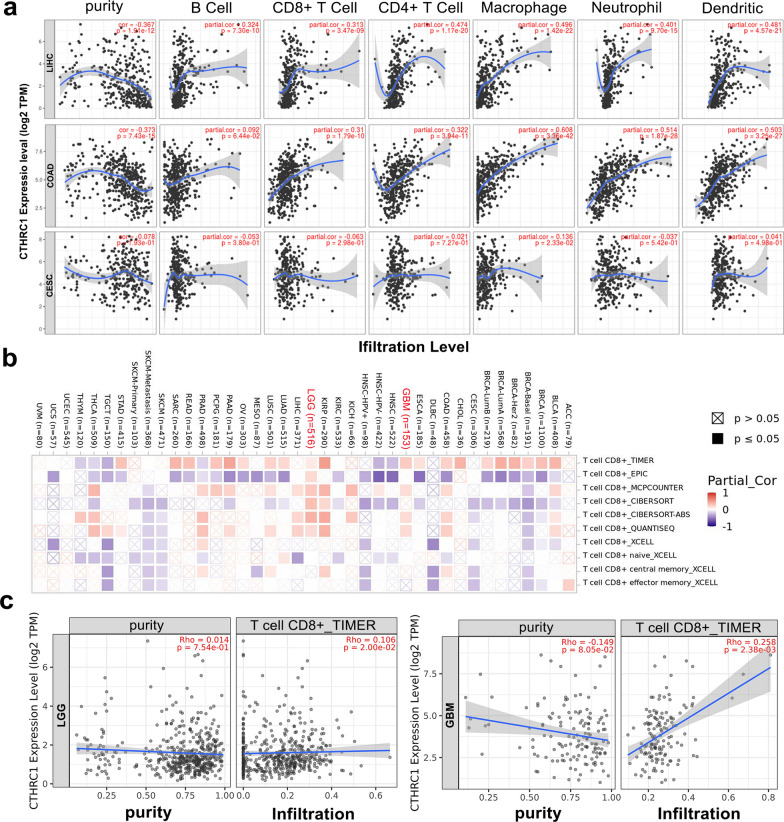
CTHRC1 expression and immune infiltration. **a** Correlation of CTHRC1 expression with immune infiltration level in LIHC, COAD, and CESC; **b** Different algorithms were used to explore the potential correlation between the expression level of CTHRC1 gene and the infiltration level of CD8 + T cells across all types of cancer in TCGA; **c** TIMER algorithm was used to explore the potential correlation between the expression level of CTHRC1 gene and the infiltration level of CD8 + T cells in LGG and GBM

For instance, the CTHRC1 expression level was linked to high level of immune cell infiltration in LIHC and COAD (Fig. 6a) and a poor prognosis (Additional file 5: Fig. S5a). Positive correlations between the CTHRC1 expression level and levels of infiltrating CD8 + T cells (r = 0.313, P = 3.47e-09), CD4 + T cells (r = 0.474, P = 1.17e-20), macrophages (r = 0.496, P = 1.42e-22), neutrophils (r = 0.401, P = 9.7e-15) and DCs (r = 0.481, P = 4.57e-21) were observed in LIHC. We also observed similar results in COAD cohorts. However, CTHRC1 expression had no significant correlations with tumor purity or levels of infiltrating B cells, CD8 + T cells, CD4 + T cells, neutrophils, and dendritic cells in CESC (Fig. 6a). The relationship between CTHRC1 expression in other cancers and different immune infiltrating cells is shown Additional file 9: Fig. S9. The analysis of the SangerBox database yielded similar results, and detailed results are shown in Additional file 10: Fig. S10a. Thus, the pattern of correlations between CTHRC1 expression and immune cell infiltration was distinctly distinguished across cancers.

We then examined the potential correlations between CTHRC1 gene expression and the level of infiltration of different immune cells in various cancers using the TIMER2 portal. Based on all or most algorithms, CTHRC1 expression negatively correlated with the immune infiltration of CD8 + T cells in CESC, DLBC, HNSC, HNSC-HPV + , KIRC, SKCM, and SKCM metastasis (Fig. 6b). A positive correlation between CTHRC1 expression and the immune infiltration of CD8 + T cells in LGG and GBM was also observed (Fig. 6b–c).

As predictors of the therapeutic efficacy of tumor immunotherapy, antitumor immunity is correlated with MSI in the tumor microenvironment [43]. We subsequently examined the relationships between MSI and CTHRC1 expression to further investigate whether CTHRC1 affects the immune mechanism and response of the TME. The results revealed that CTHRC1 gene expression was positively correlated with MSI in TGCT case (P = 8.7e-05) but was negatively linked to MSI in UCEC (P = 0.0034) (Additional file 10: Fig. S10b). Additionally, significant positive correlations were observed between these three ESTIMATE scores (ESTIMATE Score, Immune Score, and Stromal Score) and CTHRC1 expression in OV, LUSC, PRAD, BLCA, PAAD, KIRP, LIHC, BRCA, COAD, THCA, READ, LGG, KICH and PCPG (Additional file 10: Fig. S10c). Furthermore, CTHRC1 may affect antitumor immunity by regulating the components and immune mechanism in the TME.

ICP genes play an important role in immune cell infiltration and immunotherapy [44]. Therefore, we investigated the correlations between ICP gene expression and CTHRC1 expression in tumors. The results showed strong positive relationships between the expression of different ICP genes and CTHRC1 expression in many cancers, such as GBM, LGG, LUAD, etc. (Additional file 10: Fig. S10d). In particular, 32 of 47 ICP genes expressed in LIHC were linked to CTHRC1 expression. Therefore, CTHRC1 might coordinate the activation of ICP genes in diverse signal transduction pathways and represent a promising immunotherapy target. In other words, the satisfying outcome of immunotherapy targeting ICP genes might depend on high CTHRC1 expression. However, CTHRC1 was negatively correlated with the expression of ICP genes in TGCT, suggesting that patients with TGCT presenting high CTHRC1 expression respond poorly to immunotherapy targeting ICP genes.

We continued to examine the relationship between CTHRC1 expression and immune cells. As shown in Additional file 10: Fig. S10e, CTHRC1 expression was closely related to a variety of immune-related cells (activated CD4/CD8 T cells, central memory CD4/CD8 T cells, effector memory CD4/CD8 T cells, gamma delta T cells, immature B cells, macrophages, memory B cells, natural killer cells, natural killer T cells, etc.) in multiple tumors. Therefore, we summarized that CTHRC1 might serve as an ideal immunotherapy target and a predictor of the immunotherapy response.

### Analysis of the biological functions and signaling pathways of CTHRC1-related genes

**Fig. 7:**
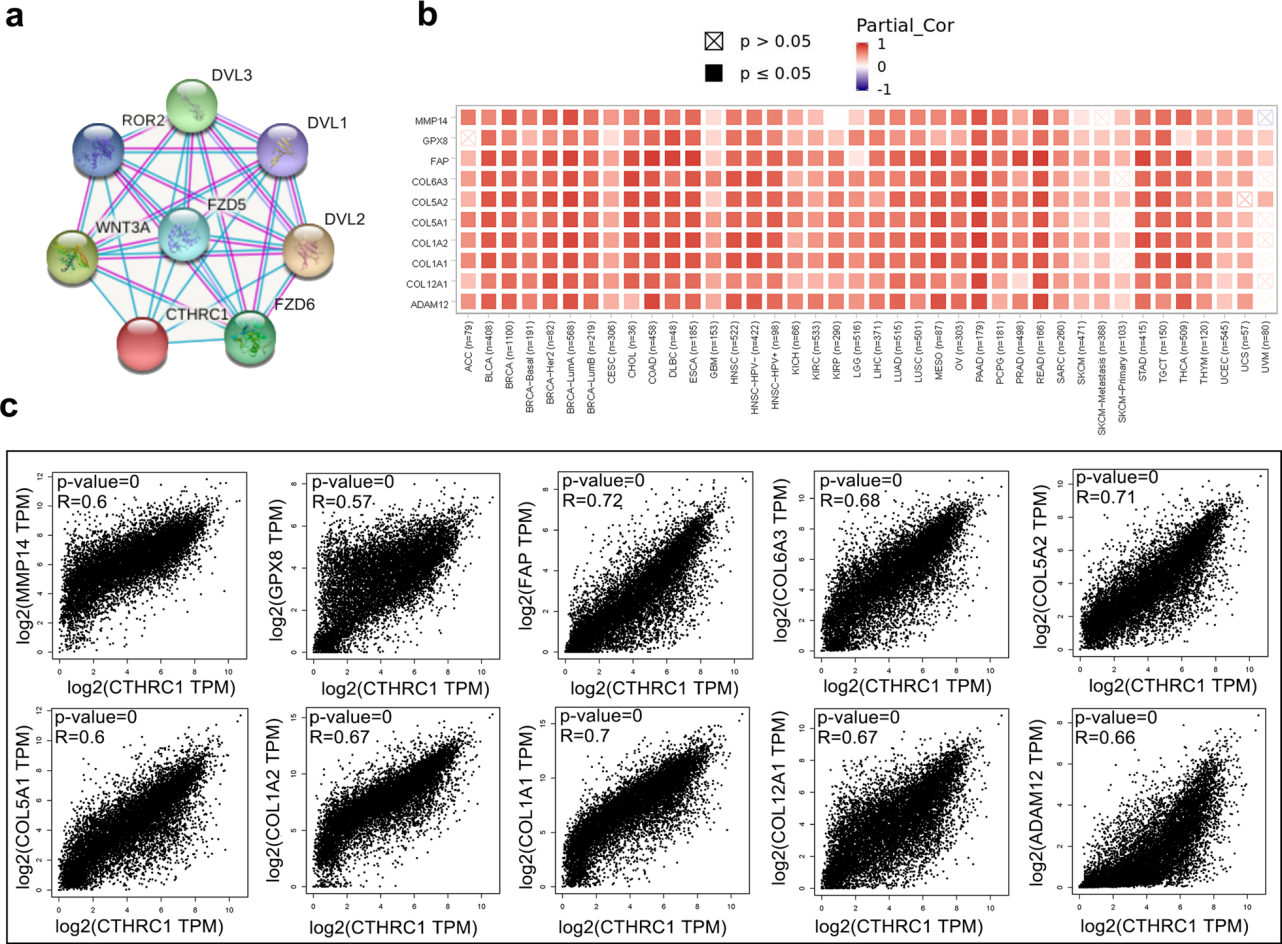
CTHRC1-related gene enrichment analysis. **a** CTHRC1-binding proteins obtained by using the STRING tool; **b** The corresponding heatmap data in the detailed cancer types are displayed. The partial correlation (cor) and P-value was generated via the purity-adjusted Spearman's rank correlation test; **c** Using the GEPIA2 approach, we also obtained the top 10 CTHRC1-correlated genes in TCGA projects and analyzed the expression correlation between CTHRC1 and selected targeting genes

Using the STRING tool, we screened the CTHRC1-binding proteins to identify the potential role of the CTHRC1 gene in tumor pathogenesis. We first screened 7 CTHRC1-binding proteins based on experimental and dataset evidence. The interaction network of FZD6, FZD5, WNT3A, ROR2, DVL1, DVL2 and DVL3 is displayed in Fig. 7a. Then, we obtained the top 10 genes that positively correlated with CTHRC1 expression using the GEPIA2 tool. The CTHRC1 gene was positively correlated with the expression of these 10 genes in most cancers and the results are displayed in the corresponding heatmap (Fig. 7b, c). To be specific, CTHRC1 expression was positively correlated with MMP14 (R = 0.60), GPX8 (R = 0.57), FAP (R = 0.72), COL6A3 (R = 0.68), COL5A2 (R = 0.71), COL5A1 (R = 0.67), COL1A2 (R = 0.70), COL1A1 (R = 0.66), COL12A1 (R = 0.67), and ADAM12 (R = 0.66) expression (all P < 0.001).

Using the LinkedOmics online database, we analyzed the genes associated with CTHRC1 in glioma (LGG/GBM), as well as the pathways and functions involved. The heatmap shows the top 50 significant genes that were positively and negatively correlated with CTHRC1 (Additional file 11: Fig. S11a, b). KEGG pathway analysis suggested enrichment in the ECM-receptor interaction, allograft rejection, autoimmune thyroid disease, *Staphylococcus aureus* infection, complement and coagulation cascades, phosphatidylinositol signaling system, glutamatergic synapse, butanoate metabolism, and other pathways (Additional file 11: Fig. S11c). Based on the GSEA function module, GO_BP term annotation revealed that CTHRC1 coexpressed genes were primarily involved in collagen metabolic process, extracellular structure organization, neutrophil mediated immunity, protein trimerization, cellular defense response, and T cell activation, while activities such as glutamate receptor signaling pathway, synaptic vesicle cycle, protein dealkylation, and RNA polyadenylation were inhibited (Additional file 11: Fig. S11d).

### Experimental identification of CTHRC1 expression levels in glioma tissue samples and cell lines

**Fig. 8:**
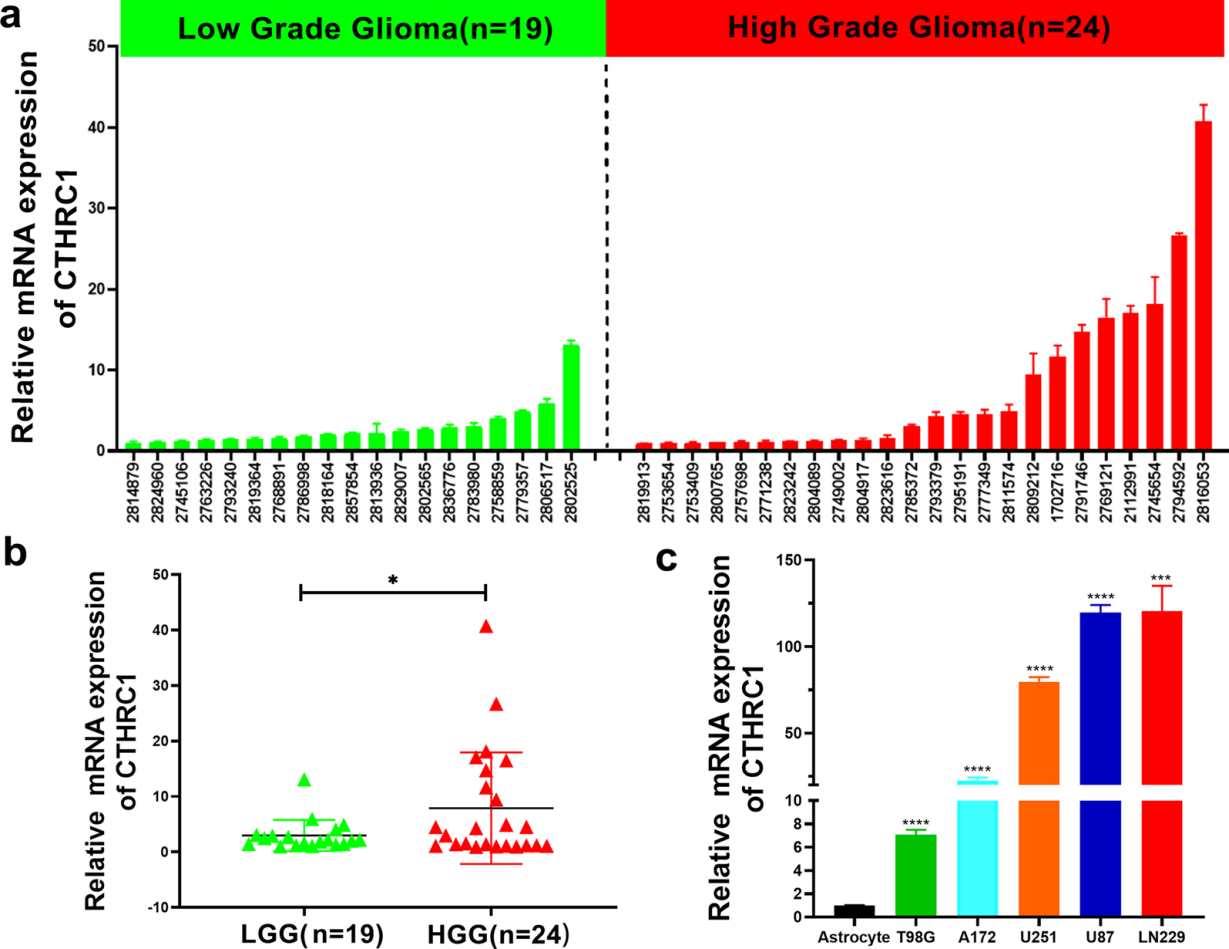
The expression of CTHRC1 in glioma tissues and cell lines. **a**, **b** CTHRC1 mRNA expression levels were detected in 43 glioma cases. **c** Expression of CTHRC1 was determined in human astrocyte and GBM cell lines

CTHRC1 expression in glioma was detected using qPCR to further investigate the level of CTHRC1 expression and its function in glioma. In addition to the online analysis, we performed qPCR experiments to validate the expression of CTHRC1 in glioma clinical tissue samples and cell lines. First, CTHRC1 was expressed at significantly higher levels in HGG tissue samples (n = 24) than in LGG tissue samples (n = 19) (Fig. 8a, b). Similarly, compared with the normal astrocyte cell line, the CTHRC1 expression level was also increased in glioma cell lines, including U87, LN229, U251, A172, and T98G (Fig. 8c). Among these cell lines, the highest CTHRC1 expression was detected in the LN229 and U87 cell lines, which might be used as cell lines for further study. Our experimental data further confirmed the reliability of the oncogenic role of CTHRC1 in glioma. The effect of CTHRC1 on the glioma phenotype requires further experimental identification.

## Discussion

As an important component of the Wnt signaling pathway, CTHRC1 has been suggested to be involved in the biogenesis and progression of various cancers and is distinctly linked to the immune cell infiltration [13, 15–17, 45, 46]. To date, few publications have provided pan-cancer insights into CTHRC1 from a holistic perspective of tumors. Therefore, we comprehensively analyzed the molecular features of CTHRC1, such as RNA and protein expression, prognosis, genetic alteration, methylation level, immunology, and relevant signaling pathways in different tumors.

We first explored CTHRC1 expression levels and found that CTHRC1 mRNA was overexpressed in most tumors, compared with adjacent normal tissues (Fig. 1c–e). In addition, CTHRC1 mRNA expression was also positively correlated with the stages of certain cancers like ACC, ESCA, KIRC, BLCA, etc. (Additional file 2: Fig. S2c). Then, our analysis showed that CTHRC1 protein, localized in the nucleus (Fig. 2d, e), was also increased in certain tumors, such as ovarian cancer, UCEC, etc. (Additional file 4: Fig. S4a, b). Based on the pan-cancer analysis, CTHRC1 mRNA and protein levels were linked to prognosis of patients with various cancers (Fig. 3, Additional file 4: Fig. S4a, Additional file 5 and 6: Figs. S5 and S6). Taken together, these results reveal that CTHRC1 is an oncogene and an important prognostic factor in some tumors, which is mutually corroborated by recent publications [16, 47–51].

It is widely accepted that cancers are triggered by gene mutations [52]. Orloff et al. reported that CTHRC1 mutations correlated with Barrett’s esophagus and esophageal adenocarcinoma [53]. TCGA data indicated that CTHRC1 gene is altered at different sites in different cancers, and the dominant alteration of CTHRC1 gene is amplification in most cancer types (Fig. 4a, b). More importantly, this gene alteration affects the prognosis of patients with tumors (Fig. 4c, d). Further studies are needed to investigate the effects of CTHRC1 gene alterations on tumorigenesis, development and prognosis.

Aberrant DNA methylation is associated with oncogenesis [38–40, 54]. Generally, less methylation at CpGs is observed in cancer cells than in normal cells [55]. A plethora of cancer-related cellular pathways are enhanced by hypomethylation of TSGs promoters, such as the Wnt signaling pathway [39, 54, 56, 57]. Interestingly, we found that CTHRC1 methylation not only plays a procancer role but also functions as a tumor suppressor in some cancers (Fig. 5a–c). CTHRC1 mRNA expression was negatively associated with the DNA methylation level in glioma (Fig. 5d). The methylation level of the CTHRC1 promoter in GBM was lower than that in LGG, suggesting that abnormal methylation of DNA promoter may be an important factor for the high expression of CTHRC1 mRNA in glioma (Fig. 5e).

The role of immunity in tumorigenesis has been widely identified [58–60]. It has been reported that CTHRC1 can regulate immune cells to mediate the development and progression of cancers, including colorectal [16], ovarian [61], endometrial [62]and pancreatic cancer [63]. In this study, our results further imply that CTHRC1 is associated with immune cell infiltration in various cancers (Fig. 6, Additional file 9, Fig. S9). We also presented evidence of the potential correlation between CTHRC1 expression and MSI, ICP and immune cells across all TCGA tumors (Additional file 10: Fig. S10). Notably, the expression of CTHRC1 was positively correlated with ICP gene CD276, which was identified a promising therapeutic target for malignant tumors (Additional file 10: Fig. S10d) [64]. These results may indicate the underlying mechanisms for CTHRC1 regulation of immune cell function in tumors.

Finally, to analyze the function of CTHRC1 in cancers, an enrichment analysis of CTHRC1-related genes and proteins was performed. We obtained 7 CTHRC1-binding proteins and constructed their interaction network (Fig. 7a). Then, we identified 10 genes which were positive correlated with the expression of CTHRC1 in most cancers (Fig. 7b, c). To further analyze the function of CTHRC1 in glioma, the top 50 genes that were positively or negatively associated with CTHRC1 expression in glioma were explored (Additional file 11: Fig. S11). KEGG and GO analyses revealed that these genes were involved in diverse pathophysiological processes, such as ECM-receptor interactions, neutrophil-mediated immunity, and T cell activation, etc. This result is consistent with our previous analysis that CTHRC1 may be associated with immunity.

Importantly, we investigated the expression of CTHRC1 in glioma clinical tissues and cell lines using qPCR. We confirmed that CTHRC1 was expressed at significantly higher levels in HGG than in LGG (Fig. 8a, b). At the same time, CTHRC1 was also proven to be expressed at high levels in glioma cell lines compared with normal astrocyte cells (Fig. 8c), suggesting they might function as oncogenes in gliomas.

There still remains many limitations in this study. Firstly, this study only verified the expression of CTHRC1 in glioma tissues and cells, but not in other tumors. Secondly, there are several conclusions based on just one analysis and a single database, and more methods or databases are needed to fully demonstrate the molecular function of CTHRC1. Thirdly, this article lacks the detailed molecular mechanisms of CTHRC1 in tumors, including glioma. Therefore, follow-ups of functional mechanisms of CTHRC1 in cancers is worth further investigating.

## Conclusions

Collectively, our pan-cancer analysis of CTHRC1 first explored the mRNA and protein expression levels, clinical prognosis, gene alterations, DNA methylation levels, immune cells infiltration, and enrichment analyses of CTHRC1, which is beneficial for understanding the function of CTHRC1 in tumorigenesis and development from diverse perspectives. We hope to identify the key targets and regulatory pathways of CTHRC1 and provide a theoretical basis for subsequent molecular targeted therapy.

## Supplementary Information

**Additional file 1: Figure S1.** Analysis process and data processing of CTHRC1 in 7 steps.

**Additional file 2: Figure S2. **CTHRC1 expression levels in different normal cells, cancer cells and pathological stages of various cancers. We analyzed the expression of the CTHRC1 mRNA in different normal cells **a** or in different cancer cells (**b**); **c** Expression levels of the CTHRC1 mRNA in different pathological stages of ACC, BLCA, ESCA, KICH, KIRC, KIRP, LUSC, PAAD, STAD, THCA.

**Additional file 3: Figure S3.** CTHRC1 mRNA expression in glioma of different grades and a single-gene GO analysis of CTHRC1. **a** The expression levels of CTHRC1 mRNA were analyzed in normal brain and glioma tissues of different grades from GTEx and TCGA databases; **b** The expression levels of CTHRC1 mRNA were analyzed in glioma of different grades in CGGA cohort (mRNA_array_301, mRNAseq_325 and mRNAseq_693 data); (c) CTHRC1 expression is positively associated with each analysis result.

**Additional file 4: Figure S4.** The immunohistochemical staining, protein expression level and prognosis of CTHRC1 protein in different tumors. **a** Immunohistochemical staining of CTHRC1 protein in renal, liver, colorectal, prostate, lung and breast cancers, and correlation between CTHRC1 protein expression and survival prognosis of these cancers. **b** The level of CTHRC1 protein is higher in ovarian, breast and colon cancers, and clear cell RCC, UCEC, LUAD than in adjacent normal tissues. Z-values represent standard deviations from the median across samples for the given cancer type. Log2 Spectral count ratio values from CPTAC were firstly normalized within each sample profile, then normalized across samples.

**Additional file 5: Figure S5.** Correlation between CTHRC1 mRNA expression and survival prognosis of cancers in TCGA. We used the GEPIA2 tool to perform overall survival **a** and disease-free survival **b** analyses of different tumors in TCGA by CTHRC1 gene expression. The survival map and Kaplan–Meier curves with positive results are given.

**Additional file 6: Figure S6.** Correlation between CTHRC1 mRNA expression and survival prognosis. The relationships between CTHRC1 expression and DFI **a** or DSS **b** prognosis of different cancers in “Gene-KM plotter” module of SangerBox; (c) A Kaplan–Meier survival curve was used to examine the expression of CTHRC1 on the all WHO grade primary glioma, WHO grade III, WHO grade IV primary glioma and HGG primary glioma survival in the CGGA cohort (mRNAseq_325, mRNAseq_693 data).

**Additional file 7: Figure S7. **CTHRC1 mutation analysis. The main mutation type **a** and mutation sites **b** of CTHRC1 were analyzed via COMIC database. **c** The primary SNV class type was G > A.

**Additional file 8: Figure S8. **CTHRC1 alteration in UCEC. **a** Lollipop plot displaying mutation distribution and protein domains for CTHRC1 in cancer with the labeled recurrent hotspots. Somatic mutation rate and transcript names are indicated by plot title and subtitle, respectively. **b** Oncoplot displaying the somatic landscape of UCEC cohort. Genes are ordered by their mutation frequency, and samples are ordered according to disease histology as indicated by the annotation bar (bottom). Side bar plot shows log10 transformed Q-values estimated by MutSigCV. Landscape of mutation profiles in UCEC samples. Mutation information of each gene in each sample was shown in the waterfall plot, where different colors with specific annotations at the bottom meant the various mutation types. The barplot above the legend exhibited the number of mutation burden. According to UCEC samples, cohort summary plot displaying distribution of variants according to variant classification (**c**), variant classification type **d**) and SNV class **e**. Bottom part indicates mutation load for each sample (**f**), variant type (**g**); A stacked barplot shows top ten mutated genes (**h**).

**Additional file 9: Figure S9.** Correlation of CTHRC1 expression with immune infiltration level in diverse type cancers via TIMER database. Gene expression levels against tumor purity is displayed on the left-most panel. The correlation of CTHRC1 expression with the abundance of immune infiltrates, including B cells, CD4 + T cells, CD8 + T cells, neutrophils, macrophages, and dendritic cells is displayed on the other panels.

**Additional file 10: Figure S10. **Gene-immune analysis of CTHCR1. The relationship between CTHRC1 expression and infiltrating levels of B cells, CD4 + T cells, CB8 + T cells, macrophages, neutrophils, dendritic cell (**a**), MSI (**b**), ESTIMATE score (**c**), ICP genes (**d**); and Immune cells (**e**) in human cancers. * P < 0.05; ** P < 0.01; *** P < 0.001.

**Additional file 11: Figure S11. **CTHRC1 coexpression genes in glioma from LinkedOmics. Heat maps showing top 50 genes positively **a** and negatively **b** correlated with CTHRC1 in glioma. Significantly enriched KEGG pathways **c** and GO annotations **d** of CTHRC1 coexpression genes in glioma.

**Additional file 12: Table S1.** Clinical data of glioma patients. **Table S2.** CTHRC1 expression in cancers and normal tissue in Oncomine database. **Table S3.** CTHRC1 single-gene GO analysis in SangerBox database.

**Additional file 13.** CTHRC1 alteration details of cancer type, protein change, mutation type.

## Data Availability

The datasets generated and/or analyzed during the current study are available in the [TCGA] repository [https://tcgadata.nci.nih.gov/tcga/] [65] and [CCLE] repository [https://sites.broadinstitute.org/ccle/] [66]. The datasets used and/or analyzed during the current study are available from the corresponding author on reasonable request.

## Abbreviations

OV: Ovarian serous cystadenocarcinoma
UCEC: Uterine corpus endometrial carcinoma
ICP: Immune checkpoint
TME: Tumor microenvironment
PCP pathway: Planar cell polarity pathway
LGG: Brain lower grade glioma
HGG: Brain higher grade glioma
ICC-IF: Immunocytochemical/immunofluorescence
RCC: Renal cell carcinoma
OS: Overall survival
DFS: Disease-free survival
DSS: Disease-specific survival
RFS: Relapse-free survival
DMFS: Distant metastasis-free survival
PPS: Post-progression survival
PFS: Progress-free survival
FP: First progression
CNA: Copy number alteration
LUSC: Lung squamous cell carcinoma
BLCA: Bladder urothelial carcinoma
COAD: Colon adenocarcinoma
CECS: Cervical squamous cell carcinoma
TIICs: Tumor-infiltrating immune cells
MSI: Microsatellite instability
PCPG: Pheochromocytoma and paraganglioma
CNS: Central nervous system
BLCA: Bladder urothelial carcinoma
BRCA: Breast invasive carcinoma
CHOL: Cholangiocarcinoma
COAD: Colon adenocarcinoma
ESCA: Esophageal carcinoma
GBM: Glioblastoma multiforme
HNSC: Head and neck cancer
KIRC: Kidney renal clear cell carcinoma
KIRP: Kidney renal papillary cell carcinoma
LIHC: Liver hepatocellular carcinoma
LUAD: Lung adenocarcinoma
LUSC: Lung squamous cell carcinoma
PRAD: Prostate adenocarcinoma
READ: Rectum adenocarcinoma
STAD: Stomach adenocarcinoma
THCA: Thyroid carcinoma
ACC: Adrenocortical carcinoma
CESC: Cervical squamous cell carcinoma and endocervical adenocarcinoma
LAML: Acute myeloid leukemia
PAAD: Pancreatic adenocarcinoma
SKCM: Skin cutaneous melanoma
TGCT: Testicular germ cell tumors
UCS: Uterine carcinosarcoma
IHC: Immunohistochemistry
SNP: Single nucleotide polymorphism
INS: Insertion
DEL: Deletion
SNV: Single nucleotide variants
TSGs: Tumor suppressor genes
MMP14: Matrix metallopeptidase 14
GPX8: Glutathione peroxidase 8
FAP: Fibroblast activation protein alpha
COL6A3: Collagen type VI alpha 3 chain
COL5A2: Collagen type V alpha 2 chain
COL5A1: Collagen type V alpha 1 chain
COL1A2: Collagen type I alpha 2 chain
COL1A1: Collagen type I alpha 1 chain
COL12A1: Collagen type XII alpha 1 chain
ADAM12: ADAM metallopeptidase domain 12
