# Supplementary figures and images for: Pan-cancer analysis combined with experiments predicts CTHRC1 as a therapeutic target for human cancers
Source: Cancer Cell Int. 2021 Oct 26;21:566. doi: 10.1186/s12935-021-02266-3 (PMC8549344; doi:10.1186/s12935-021-02266-3)

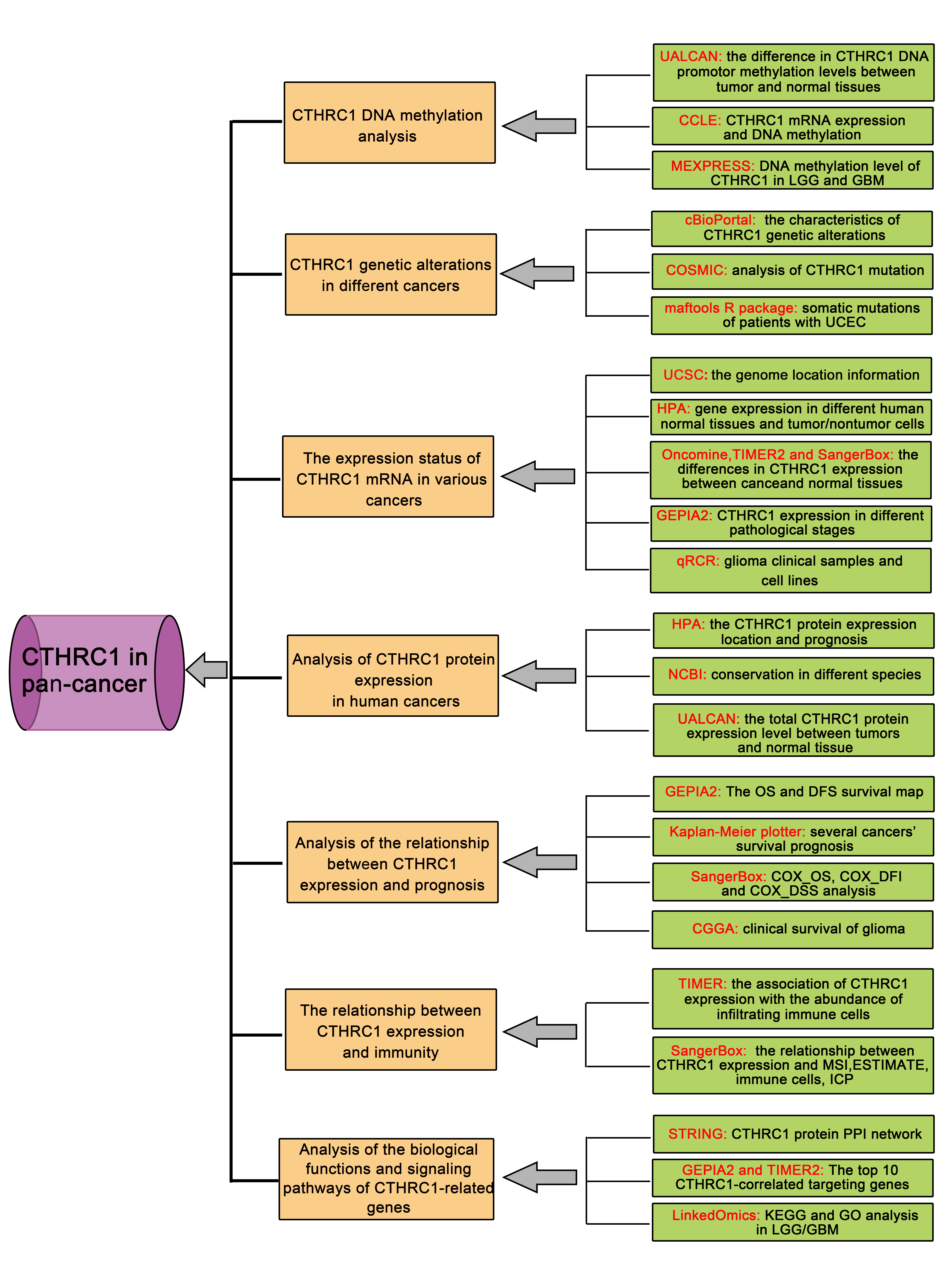

Supplement: Supplementary file 1 — Additional file 1: Figure S1. Analysis process and data processing of CTHRC1 in 7 steps. [file 12935_2021_2266_MOESM1_ESM.tif]

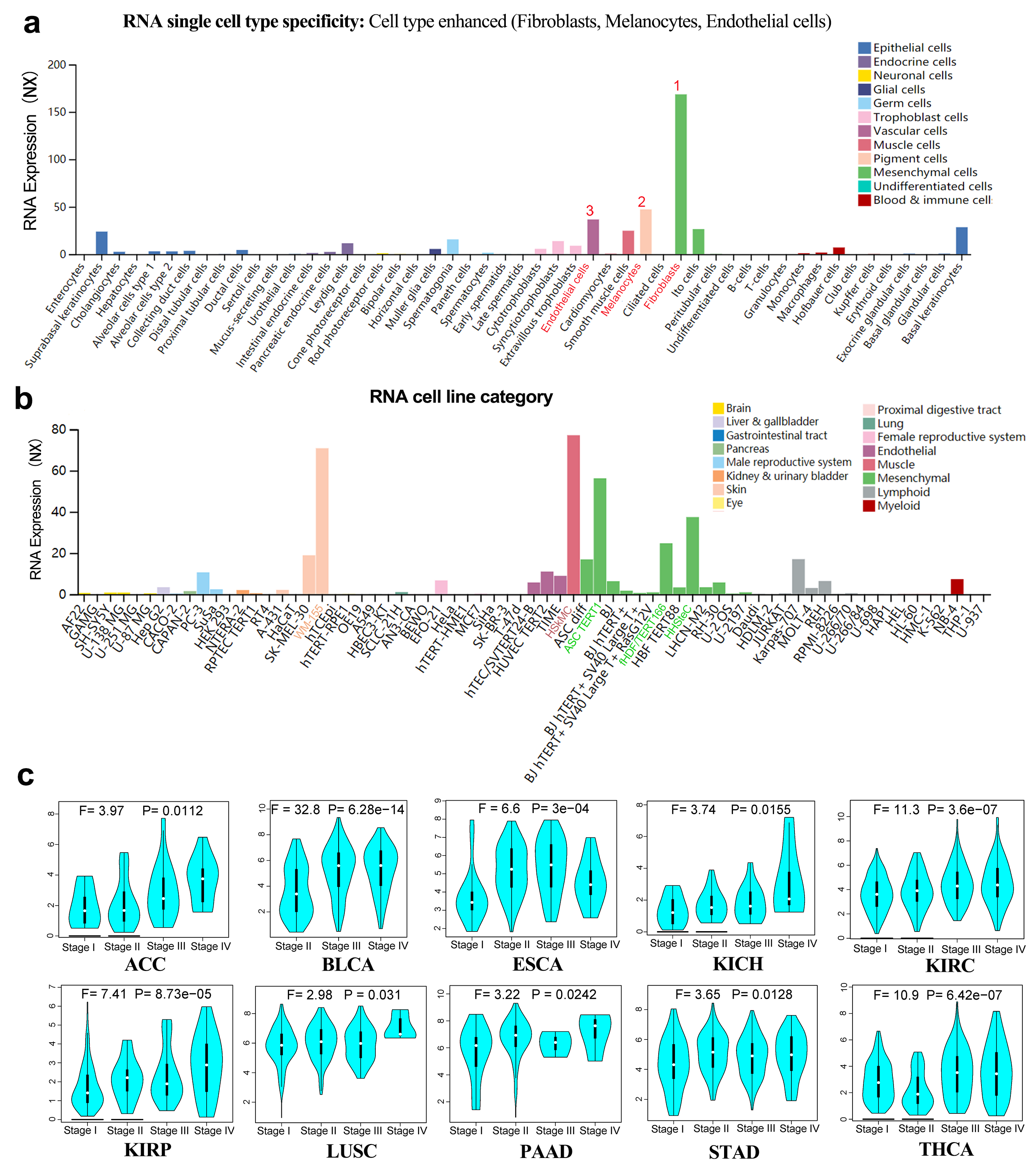

Supplement: Supplementary file 2 — Additional file 2: Figure S2. CTHRC1 expression levels in different normal cells, cancer cells and pathological stages of various cancers. We analyzed the expression of the CTHRC1 mRNA in different normal cells a or in different cancer cells (b); c Expression levels of the CTHRC1 mRNA in different pathological stages of ACC, BLCA, ESCA, KICH, KIRC, KIRP, LUSC, PAAD, STAD, THCA. [file 12935_2021_2266_MOESM2_ESM.tif]

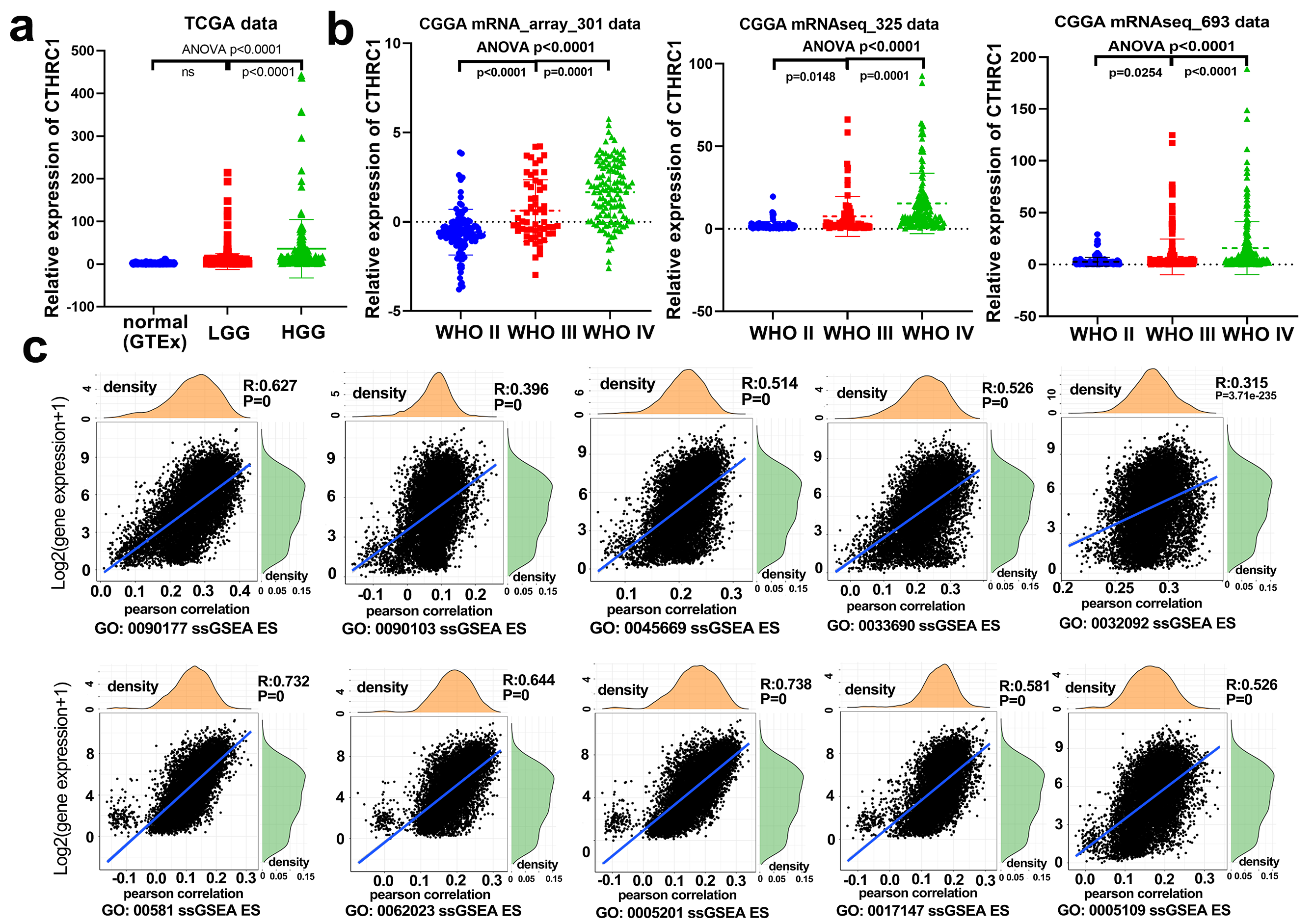

Supplement: Supplementary file 3 — Additional file 3: Figure S3. CTHRC1 mRNA expression in glioma of different grades and a single-gene GO analysis of CTHRC1. a The expression levels of CTHRC1 mRNA were analyzed in normal brain and glioma tissues of different grades from GTEx and TCGA databases; b The expression levels of CTHRC1 mRNA were analyzed in glioma of different grades in CGGA cohort (mRNA_array_301, mRNAseq_325 and mRNAseq_693 data); (c) CTHRC1 expression is positively associated with each analysis result. [file 12935_2021_2266_MOESM3_ESM.tif]

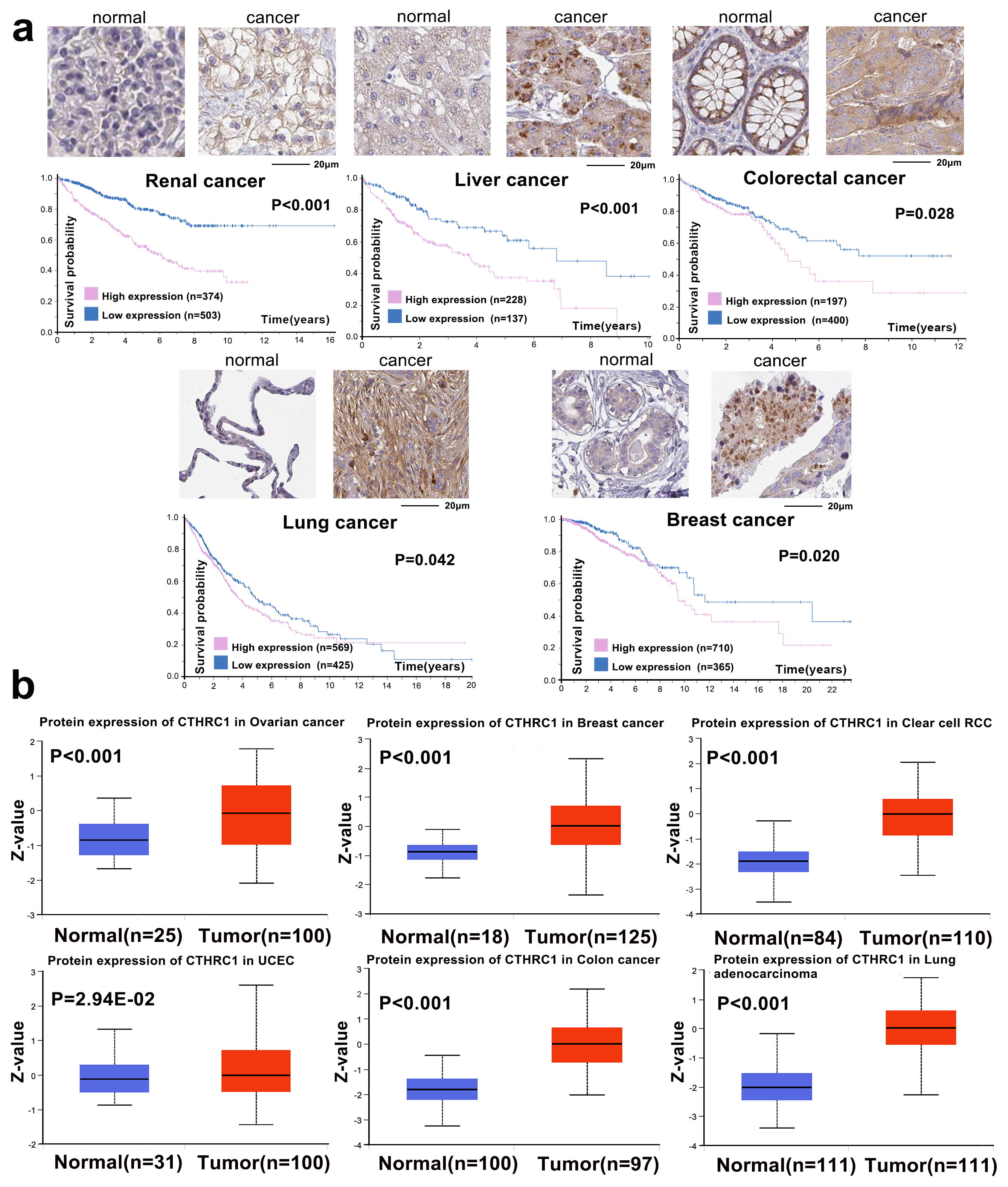

Supplement: Supplementary file 4 — Additional file 4: Figure S4. The immunohistochemical staining, protein expression level and prognosis of CTHRC1 protein in different tumors. a Immunohistochemical staining of CTHRC1 protein in renal, liver, colorectal, prostate, lung and breast cancers, and correlation between CTHRC1 protein expression and survival prognosis of these cancers. b The level of CTHRC1 protein is higher in ovarian, breast and colon cancers, and clear cell RCC, UCEC, LUAD than in adjacent normal tissues. Z-values represent standard deviations from the median across samples for the given cancer type. Log2 Spectral count ratio values from CPTAC were firstly normalized within each sample profile, then normalized across samples. [file 12935_2021_2266_MOESM4_ESM.tif]

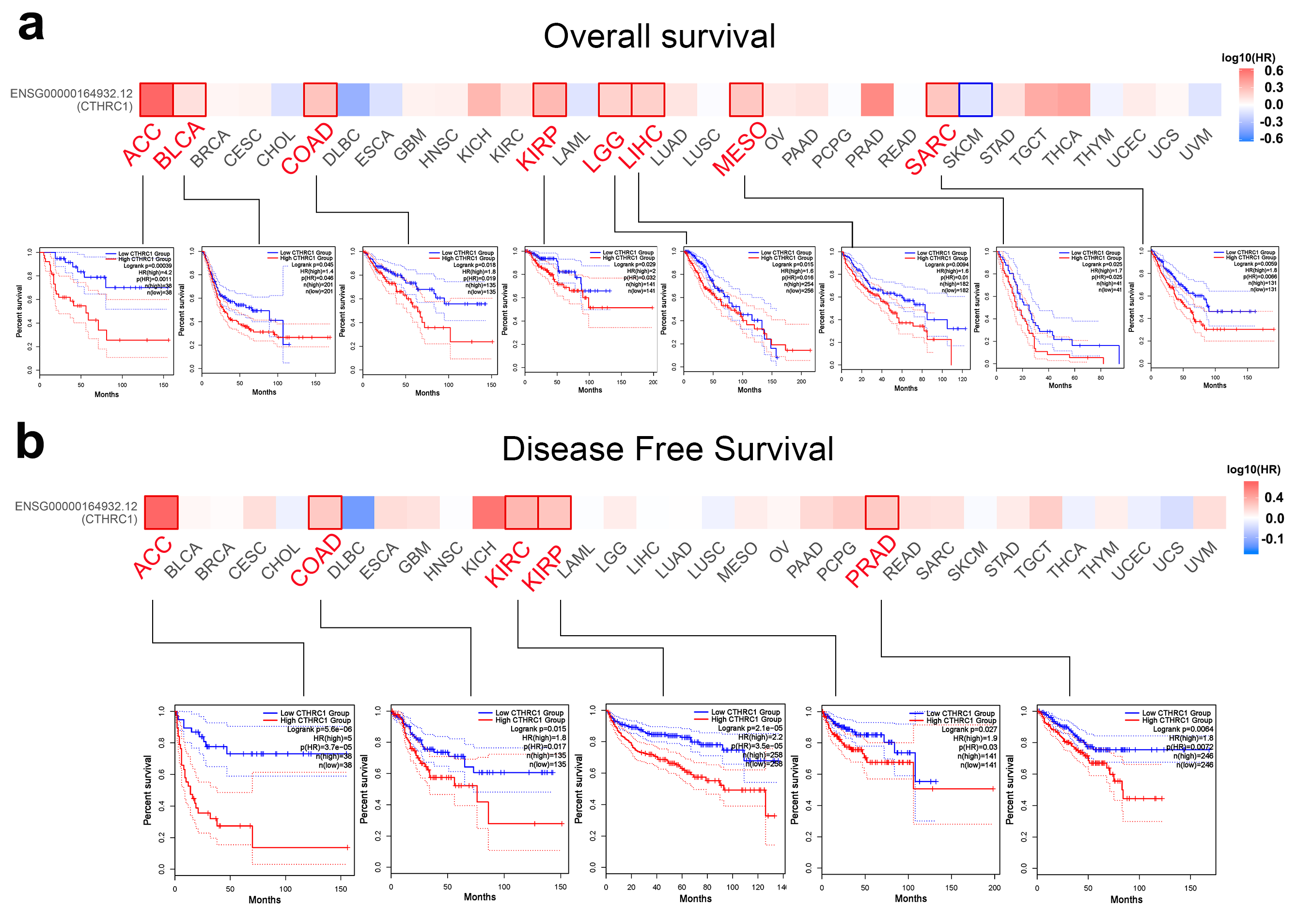

Supplement: Supplementary file 5 — Additional file 5: Figure S5. Correlation between CTHRC1 mRNA expression and survival prognosis of cancers in TCGA. We used the GEPIA2 tool to perform overall survival a and disease-free survival b analyses of different tumors in TCGA by CTHRC1 gene expression. The survival map and Kaplan–Meier curves with positive results are given. [file 12935_2021_2266_MOESM5_ESM.tif]

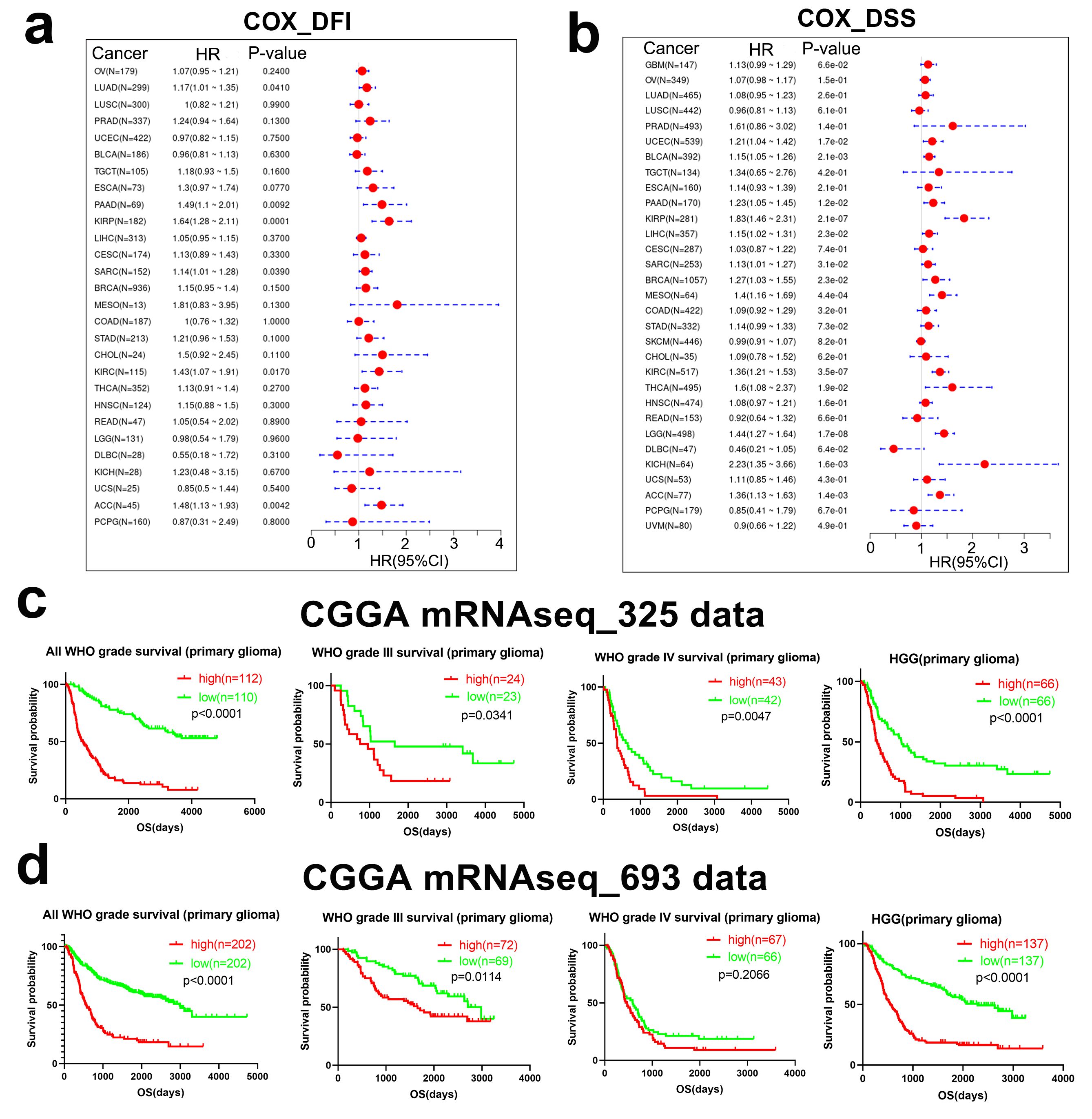

Supplement: Supplementary file 6 — Additional file 6: Figure S6. Correlation between CTHRC1 mRNA expression and survival prognosis. The relationships between CTHRC1 expression and DFI a or DSS b prognosis of different cancers in “Gene-KM plotter” module of SangerBox; (c) A Kaplan–Meier survival curve was used to examine the expression of CTHRC1 on the all WHO grade primary glioma, WHO grade III, WHO grade IV primary glioma and HGG primary glioma survival in the CGGA cohort (mRNAseq_325, mRNAseq_693 data). [file 12935_2021_2266_MOESM6_ESM.tif]

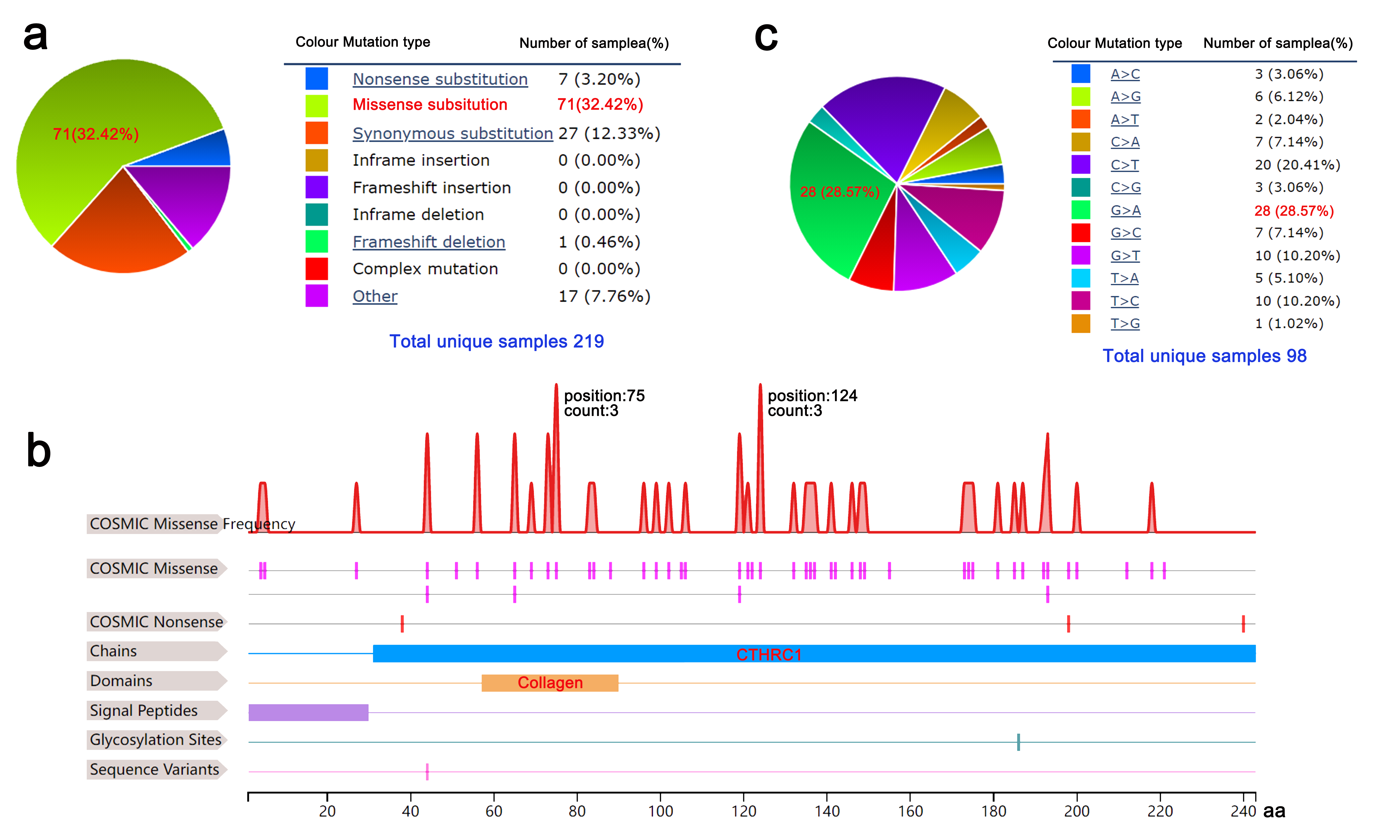

Supplement: Supplementary file 7 — Additional file 7: Figure S7. CTHRC1 mutation analysis. The main mutation type a and mutation sites b of CTHRC1 were analyzed via COMIC database. c The primary SNV class type was G > A. [file 12935_2021_2266_MOESM7_ESM.tif]

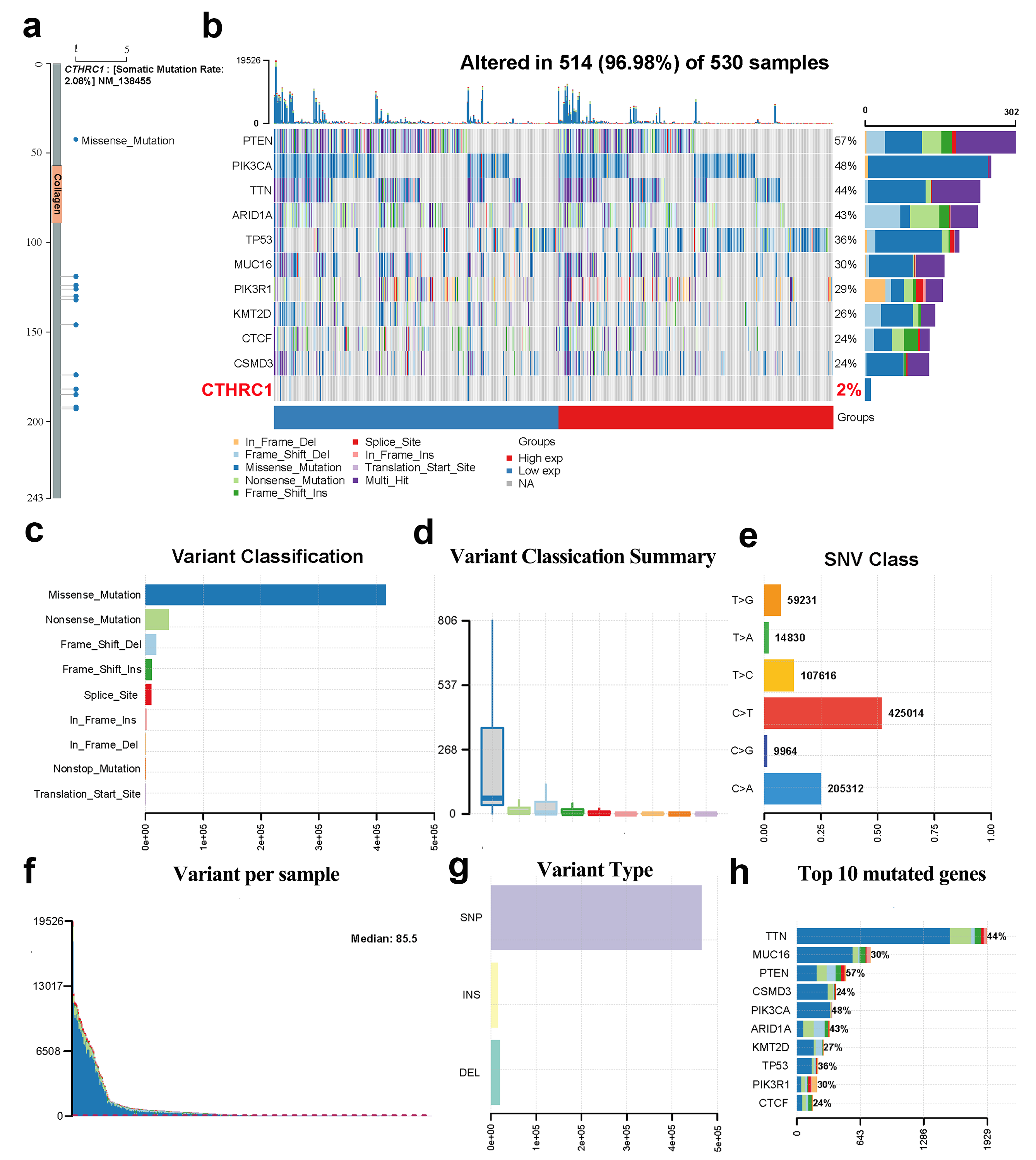

Supplement: Supplementary file 8 — Additional file 8: Figure S8. CTHRC1 alteration in UCEC. a Lollipop plot displaying mutation distribution and protein domains for CTHRC1 in cancer with the labeled recurrent hotspots. Somatic mutation rate and transcript names are indicated by plot title and subtitle, respectively. b Oncoplot displaying the somatic landscape of UCEC cohort. Genes are ordered by their mutation frequency, and samples are ordered according to disease histology as indicated by the annotation bar (bottom). Side bar plot shows log10 transformed Q-values estimated by MutSigCV. Landscape of mutation profiles in UCEC samples. Mutation information of each gene in each sample was shown in the waterfall plot, where different colors with specific annotations at the bottom meant the various mutation types. The barplot above the legend exhibited the number of mutation burden. According to UCEC samples, cohort summary plot displaying distribution of variants according to variant classification (c), variant classification type d) and SNV class e. Bottom part indicates mutation load for each sample (f), variant type (g); A stacked barplot shows top ten mutated genes (h). [file 12935_2021_2266_MOESM8_ESM.tif]

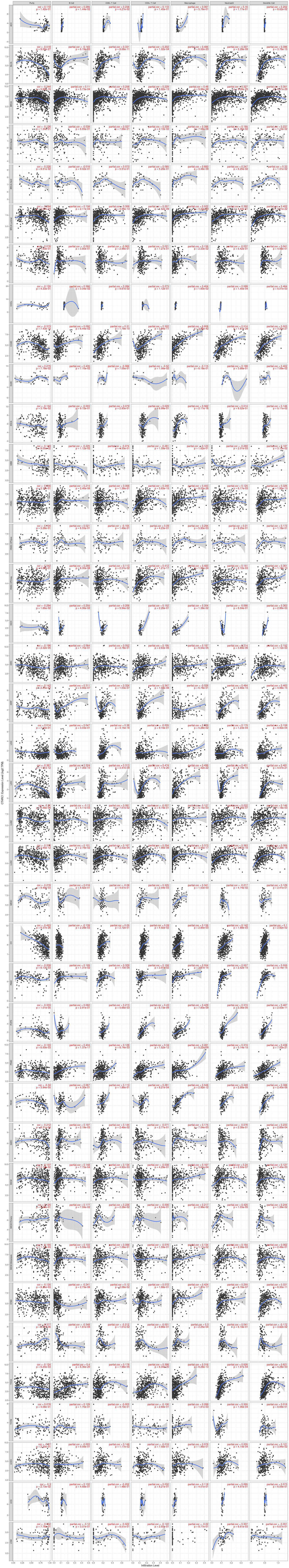

Supplement: Supplementary file 9 — Additional file 9: Figure S9. Correlation of CTHRC1 expression with immune infiltration level in diverse type cancers via TIMER database. Gene expression levels against tumor purity is displayed on the left-most panel. The correlation of CTHRC1 expression with the abundance of immune infiltrates, including B cells, CD4 + T cells, CD8 + T cells, neutrophils, macrophages, and dendritic cells is displayed on the other panels. [file 12935_2021_2266_MOESM9_ESM.tiff]

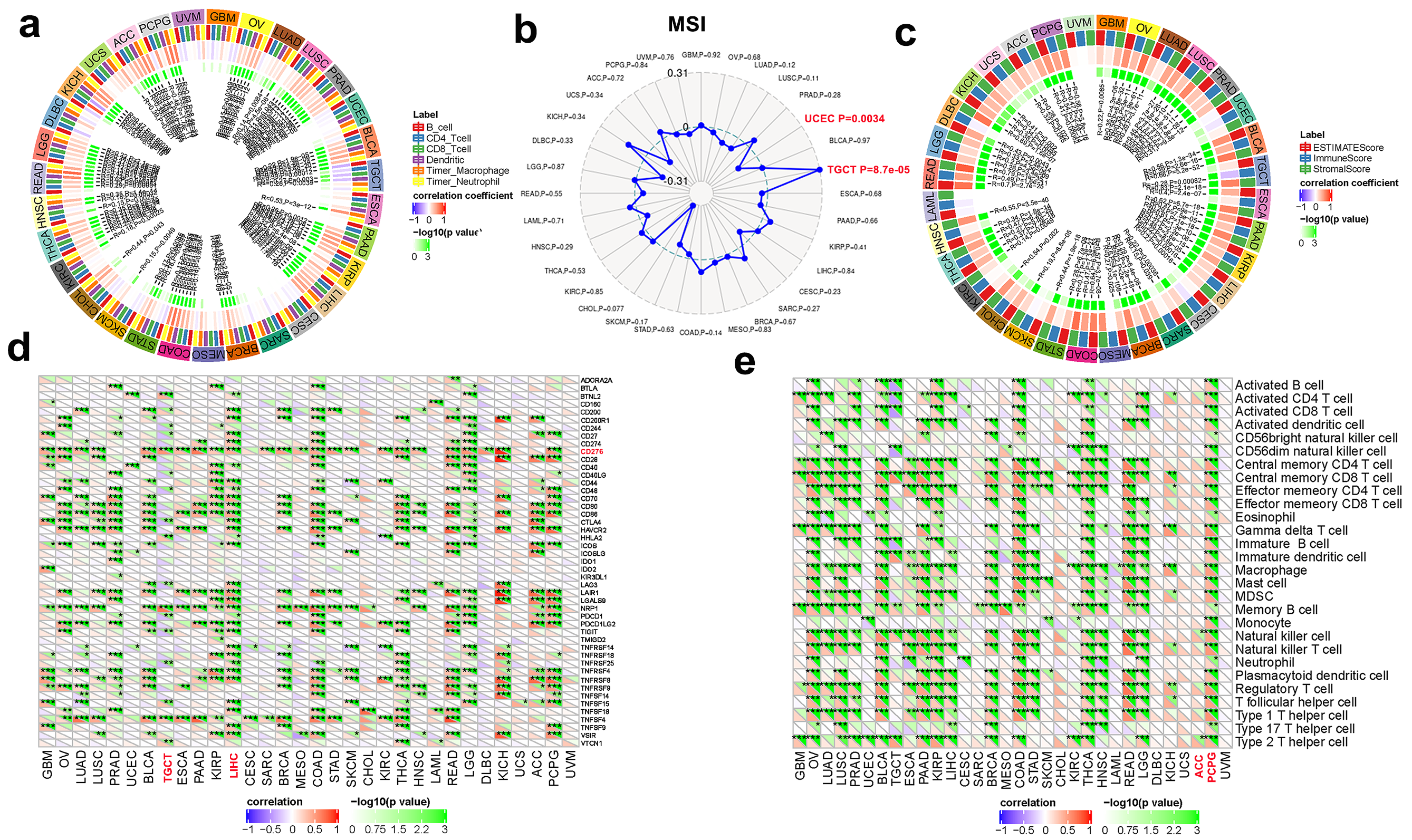

Supplement: Supplementary file 10 — Additional file 10: Figure S10. Gene-immune analysis of CTHCR1. The relationship between CTHRC1 expression and infiltrating levels of B cells, CD4 + T cells, CB8 + T cells, macrophages, neutrophils, dendritic cell (a), MSI (b), ESTIMATE score (c), ICP genes (d); and Immune cells (e) in human cancers. * P < 0.05; ** P < 0.01; *** P < 0.001. [file 12935_2021_2266_MOESM10_ESM.tif]

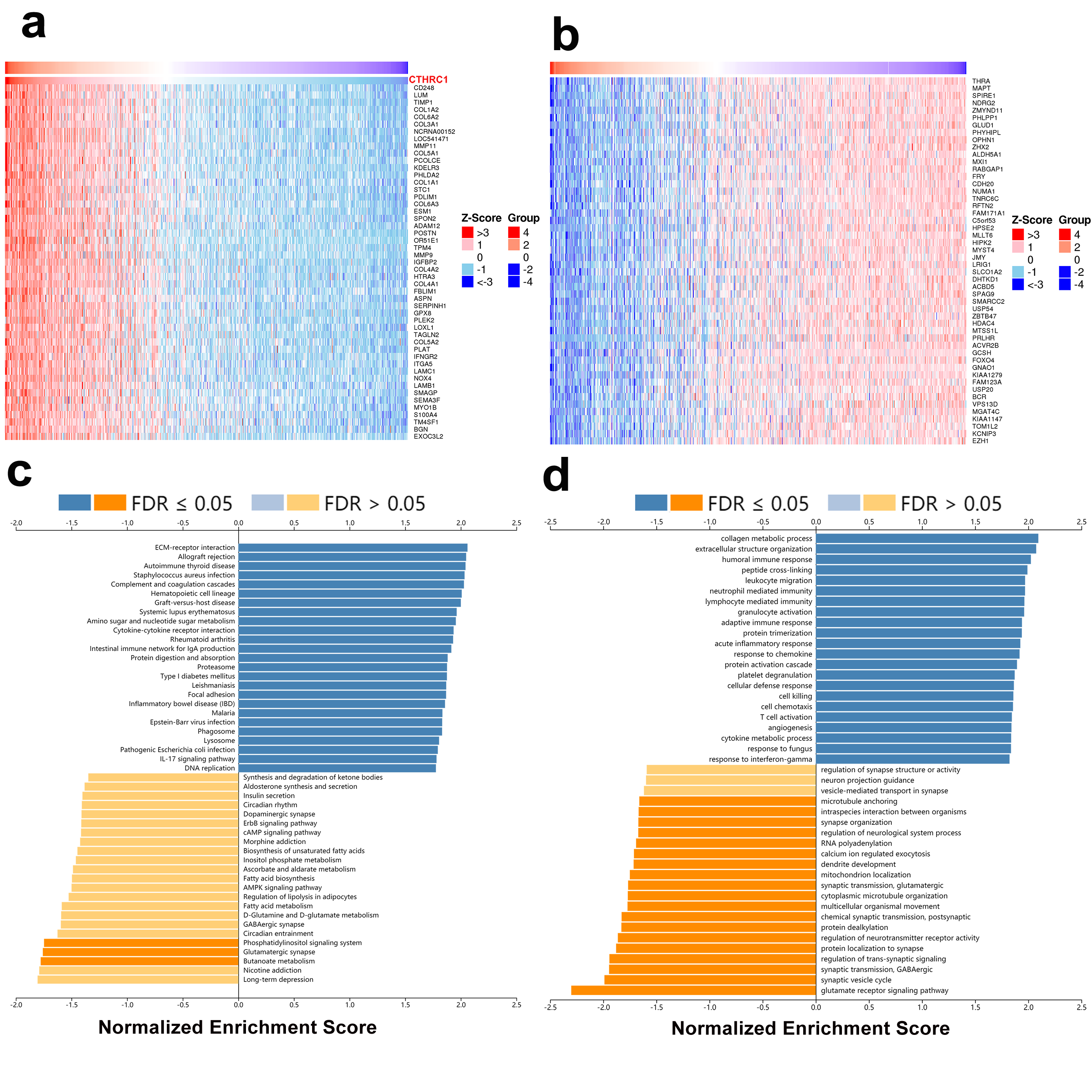

Supplement: Supplementary file 11 — Additional file 11: Figure S11. CTHRC1 coexpression genes in glioma from LinkedOmics. Heat maps showing top 50 genes positively a and negatively b correlated with CTHRC1 in glioma. Significantly enriched KEGG pathways c and GO annotations d of CTHRC1 coexpression genes in glioma. [file 12935_2021_2266_MOESM11_ESM.tif]
