# Supplementary material for: Pan-cancer analysis combined with experiments predicts CTHRC1 as a therapeutic target for human cancers
Source: Cancer Cell Int. 2021 Oct 26;21:566. doi: 10.1186/s12935-021-02266-3 (PMC8549344; doi:10.1186/s12935-021-02266-3)
Supplement: Supplementary file 12 — Additional file 12: Table S1. Clinical data of glioma patients. Table S2. CTHRC1 expression in cancers and normal tissue in Oncomine database. Table S3. CTHRC1 single-gene GO analysis in SangerBox database. [file 12935_2021_2266_MOESM12_ESM.docx]

**Table S1. Clinical data of glioma patients.**

| **Case ID** | **Grade** | **Pathology** | **Age** | **Gender** | **Location** |  |
| --- | --- | --- | --- | --- | --- | --- |
| 2814879 | Grade2 | Anaplastic-Oligodendroglioma | 62 | Female | Left-Frontal lobe |  |
| 2745106 | Grade2 | Astrocytoma | 47 | Male | Right-Temporal lobe | |
| 2763226 | Grade2 | Astrocytoma | 22 | Female | Right-Temporal lobe and Insula |  |
| 2824960 | Grade2 | Oligodendroglioma | 66 | Male | Left-Temporal lobe |  |
| 2768891 | Grade2 | Oligoastrocytoma | 46 | Male | Right-insula |  |
| 2806517 | Grade2 | Oligodendroglioma | 42 | Male | Right-Frontal lobe |  |
| 2857854 | Grade2 | Astrocytoma | 47 | Male | Right-Frontal and Temporal lobe and Insula |  |
| 2758859 | Grade2 | Astrocytoma | 54 | Female | Left-Frontal and Temporal lobe |  |
| 2779357 | Grade2 | Oligoastrocytoma | 37 | Male | Right-Temporal lobe and Insula |  |
| 2783980 | Grade2 | Astrocytoma | 26 | Male | Left-Frontal lobe and Corpus Callosum |  |
| 2802525 | Grade2 | Oligodendroglioma | 49 | Female | Both Frontal lobes |  |
| 2813936 | Grade2 | Astrocytoma | 21 | Female | Right-Frontal lobe |  |
| 2818164 | Grade2 | Astrocytoma | 37 | Male | Right-Temporal lobe、Basal ganglia and Thalamus |  |
| 2836776 | Grade2 | Oligodendroglioma | 66 | Female | Left-Frontal lobe |  |
| 2793240 | Grade2 | Astrocytoma | 53 | Female | Left-Parietal and Occipital lobe |  |
| 2786998 | Grade2 | Astrocytoma | 65 | Female | Left-Insula and Hippocampus |  |
| 2819364 | Grade2 | Oligodendroglioma | 35 | Male | Right-Frontal lobe |  |
| 2829007 | Grade2 | Astrocytoma | 13 | Male | Right-cerebellum |  |
| 2802565 | Grade2 | Oligodendroglioma | 36 | Female | Left-Frontal lobe |  |
| 2794592 | Grade3 | Anaplastic- Astrocytoma | 46 | Male | Right-Frontal and Temporal lobe and Basal ganglia |  |
| 2777349 | Grade3 | Anaplastic- Astrocytoma | 13 | Male | Left-Frontal and Parietal lobe |  |
| 2809212 | Grade3 | Anaplastic- Oligodendroglioma | 44 | Male | Left-Frontal lobe |  |
| 1702716 | Grade3 | Anaplastic- Oligodendroglioma | 65 | Male | Right-Frontal lobe |  |
| 2800765 | Grade3 | Anaplastic- Astrocytoma | 55 | Female | Right-Frontal and Parietal lobe |  |
| 2823242 | Grade3 | Anaplastic- Oligodendroglioma | 50 | Female | Left-Frontal lobe |  |
| 2804917 | Grade3 | Anaplastic- Oligodendroglioma | 48 | Female | Right-Frontal lobe |  |
| 2819913 | Grade3 | Oligodendroglioma & Anaplastic- Oligodendroglioma | 29 | Female | Right-Frontal and Parietal lobe |  |
| 2804089 | Grade3 | Astrocytoma & Anaplastic- Astrocytoma | 43 | Female | Right-Temporal lobe |  |
| 2785372 | Grade4 | Anaplastic- Astrocytoma & Glioblastoma | 77 | Male | Right-Frontal and Parietal lobe |  |
| 2791746 | Grade4 | Glioblastoma | 69 | Male | Right-Temporal lobe |  |
| 2112991 | Grade4 | Glioblastoma | 52 | Female | Right-Temporal lobe |  |
| 2771238 | Grade4 | Glioblastoma | 69 | Female | Left-Frontal lobe |  |
| 2823616 | Grade4 | Glioblastoma | 54 | Male | Left-Frontal lobe |  |
| 2753654 | Grade4 | Glioblastoma | 56 | Female | Left-Frontal lobe |  |
| 2753409 | Grade4 | Glioblastoma | 21 | Female | Right-Frontal lobe |  |
| 2757698 | Grade4 | Glioblastoma | 16 | Male | Left-Occipital lobe |  |
| 2811574 | Grade4 | Glioblastoma | 51 | Female | Right-Temporal lobe and insula |  |
| 2745654 | Grade4 | Glioblastoma | 70 | Female | Left-Frontal and Parietal lobe |  |
| 2793379 | Grade4 | Glioblastoma | 78 | Male | Right-Frontal lobe |  |
| 2816053 | Grade4 | Glioblastoma | 52 | Male | Left-Temporal lobe |  |
| 2749002 | Grade4 | Glioblastoma | 57 | Male | Both-Frontal lobes |  |
| 2769121 | Grade4 | Glioblastoma | 43 | Male | Left-Temporal lobe |  |
| 2795191 | Grade4 | Glioblastoma | 44 | Female | Left-Temporal lobe and Basal ganglia |  |

**Table S2. CTHRC1 expression in cancers and normal tissue in Oncomine database.**

| **Cancer** | **Cancer type** | **P-value** | **FC** | **Rank** | **Sample** | **Reference (PMID)** |
| --- | --- | --- | --- | --- | --- | --- |
| Bladder | Infiltrating Bladder Urothelial Carcinoma | 0.000000589 | 1.754 | 0.02 | 130 | 20421545 |
|  | Infiltrating Bladder Urothelial Carcinoma | 6.86E-10 | 2.445 | 0.04 | 129 | 16432078 |
|  | Superficial Bladder Cancer | 8.28E-12 | 2.891 | 0.09 | 76 | 16432078 |
|  |  |  |  |  |  |  |
| Brain and CNS | Glioblastoma | 0.0000119 | 2.111 | 0.18 | 104 | 16616334 |
|  | Glioblastoma | 0.0000018 | -8.289 | 0.11 | 25 | 16697959 |
|  |  |  |  |  |  |  |
| Breast | Invasive Ductal Breast Carcinoma | 8.80E-45 | 2.796 | 0.01 | 450 | TCGA |
|  | Invasive Lobular Breast Carcinoma | 8.45E-14 | 1.913 | 0.02 | 97 | TCGA |
|  | Invasive Breast Carcinoma | 3.9E-21 | 2.633 | 0.03 | 137 | TCGA |
|  | Male Breast Carcinoma | 0.00000826 | 2.34 | 0.03 | 64 | TCGA |
|  | Ductal Breast Carcinoma | 4.07E-09 | 2.721 | 0.01 | 47 | 16473279 |
|  | Invasive Ductal Breast Carcinoma | 3.82E-85 | 1.859 | 0.02 | 1700 | 22522925 |
|  | Medullary Breast Carcinoma | 4.11E-12 | 2.381 | 0.03 | 176 | 22522925 |
|  | Invasive Breast Carcinoma | 0.000000961 | 1.708 | 0.03 | 165 | 22522925 |
|  | Invasive Ductal and Invasive Lobular Breast Carcinoma | 1.18E-23 | 1.625 | 0.04 | 234 | 22522925 |
|  | Invasive Lobular Breast Carcinoma | 7.92E-26 | 1.508 | 0.06 | 292 | 22522925 |
|  | Ductal Breast Carcinoma in Situ | 0.000671 | 1.516 | 0.07 | 254 | 22522925 |
|  | Ductal Breast Carcinoma in Situ | 0.000373 | 1.818 | 0.04 | 23 | 19187537 |
|  | Lobular Breast Carcinoma | 0.000167 | 1.879 | 0.07 | 24 | 15034139 |
|  | Invasive Ductal Breast Carcinoma | 0.000902 | 1.758 | 0.14 | 41 | 15034139 |
|  | Invasive Breast Carcinoma | 0.00000708 | -3.958 | 0.31 | 59 | 18438415 |
|  |  |  |  |  |  |  |
| Cervical | Cervical Squamous Cell Carcinoma | 4.04E-08 | 2.65 | 0.02 | 31 | 17974957 |
|  | Cervical Cancer | 1.06E-09 | 2.881 | 0.02 | 42 | 17510386 |
|  | Cervical Squamous Cell Carcinoma | 0.000000469 | 2.631 | 0.04 | 56 | 18506748 |
|  | Cervical Squamous Cell Carcinoma | 0.000437 | 1.56 | 0.19 | 45 | 18191186 |
|  |  |  |  |  |  |  |
| Colorectal | Rectal Mucinous Adenocarcinoma | 0.00000453 | 1.959 | 0.03 | 28 | TCGA |
|  | Cecum Adenocarcinoma | 4.88E-09 | 2.184 | 0.07 | 44 | TCGA |
|  | Colon Mucinous Adenocarcinoma | 0.000000021 | 2.059 | 0.07 | 44 | TCGA |
|  | Rectal Adenocarcinoma | 2.53E-10 | 2.163 | 0.11 | 82 | TCGA |
|  | Colon Adenocarcinoma | 5.09E-10 | 2.129 | 0.13 | 123 | TCGA |
|  | Colon Adenoma | 3.27E-13 | 2.795 | 0.04 | 57 | 18171984 |
|  | Rectal Adenoma | 0.00000235 | 2.945 | 0.04 | 39 | 18171984 |
|  | Colon Adenocarcinoma | 5.67E-08 | 1.578 | 0.09 | 91 | 17640062 |
|  | Rectal Adenocarcinoma | 6.75E-21 | 1.899 | 0.05 | 130 | 20725992 |
|  | Colon Adenoma | 0.0000271 | 2.436 | 0.07 | 15 | 20957034 |
|  |  |  |  |  |  |  |
| Esophageal Squamous Cell Carcinoma | Esophageal Squamous Cell Carcinoma | 0.0000197 | 1.651 | 0.07 | 34 | 20955586 |
|  |  |  |  |  |  |  |
| Gastric | Gastric Mixed Adenocarcinoma | 0.0000231 | 1.894 | 0.01 | 29 | 21447720 |
|  | Gastric Adenocarcinoma | 0.000761 | 1.648 | 0.01 | 23 | 21447720 |
|  | Diffuse Gastric Adenocarcinoma | 0.000034 | 1.536 | 0.04 | 50 | 21447720 |
|  | Gastric Intestinal Type Adenocarcinoma | 8.45E-09 | 4.136 | 0.06 | 57 | 19081245 |
|  |  |  |  |  |  |  |
| Head and neck | Head and Neck Squamous Cell Carcinoma | 0.0000173 | 1.684 | 0.02 | 38 | 14676830 |
|  | Floor of the Mouth Carcinoma | 0.000000146 | 2.959 | 0.01 | 27 | 17510386 |
|  | Salivary Gland Adenoid Cystic Carcinoma | 0.000876 | 11.499 | 0.1 | 22 | 12368205 |
|  | Nasopharyngeal Carcinoma | 0.0000279 | 1.728 | 0.05 | 41 | 16912175 |
|  | Tongue Squamous Cell Carcinoma | 0.0000467 | 2.018 | 0.14 | 57 | 19138406 |
|  | Oral Cavity Squamous Cell Carcinoma | 0.0000128 | 1.612 | 0.13 | 79 | 21853135 |
|  |  |  |  |  |  |  |
| Leukemia | Acute Myeloid Leukemia | 0.00000699 | 10.272 | 0.01 | 15 | 14770183 |
|  | B-Cell Acute Lymphoblastic Leukemia | 0.000102 | -1.96 | 0.05 | 14 | 16267031 |
|  | Chronic Lymphocytic Leukemia | 0.000013 | -3.716 | 0.09 | 59 | 15778709 |
|  | T-Cell Childhood Acute Lymphoblastic Leukemia | 0.0000764 | -2.122 | 0.07 | 50 | 21487112 |
|  | B-Cell Childhood Acute Lymphoblastic Leukemia | 0.000553 | -1.975 | 0.07 | 242 | 21487112 |
|  | Chronic Lymphocytic Leukemia | 3.95E-35 | -2.505 | 0.06 | 522 | 20406941 |
|  | Pro-B Acute Lymphoblastic Leukemia | 6.22E-17 | -1.76 | 0.1 | 144 | 20406941 |
|  | B-Cell Childhood Acute Lymphoblastic Leukemia | 1.95E-18 | -1.638 | 0.12 | 433 | 20406941 |
|  |  |  |  |  |  |  |
| Liver | Hepatocellular Carcinoma | 1.03E-09 | 1.54 | 0.06 | 179 | 12058060 |
|  |  |  |  |  |  |  |
| Lung | Large Cell Lung Carcinoma | 0.000578 | 2.363 | 0.02 | 10 | 11707590 |
|  | Small Cell Lung Carcinoma | 0.000781 | 2.955 | 0.02 | 10 | 11707590 |
|  | Small Cell Lung Carcinoma | 0.000834 | 3.316 | 0.04 | 23 | 11707567 |
|  | Squamous Cell Lung Carcinoma | 1.64E-12 | 2.415 | 0.03 | 92 | 20421987 |
|  | Large Cell Lung Carcinoma | 0.000000442 | 3.104 | 0.03 | 84 | 20421987 |
|  | Lung Adenocarcinoma | 1.97E-10 | 1.968 | 0.04 | 110 | 20421987 |
|  | Lung Adenocarcinoma | 0.000165 | 2.24 | 0.07 | 57 | 17540040 |
|  | Lung Adenocarcinoma | 1.41E-10 | 1.915 | 0.07 | 246 | 22080568 |
|  |  |  |  |  |  |  |
| Lymphoma | Activated B-Cell-Like Diffuse Large B-Cell Lymphoma | 0.000000674 | 2.198 | 0.05 | 61 | 10676951 |
|  | Germinal Center B-Cell-Like Diffuse Large B-Cell Lymphoma | 0.000156 | 1.711 | 0.12 | 65 | 10676951 |
|  | Burkitt's Lymphoma | 0.000525 | 2.15 | 0.17 | 42 | 15778709 |
|  | Unspecified Peripheral T-Cell Lymphoma | 8.7E-09 | 2.728 | 0.09 | 48 | 17304354 |
|  | Angioimmunoblastic T-Cell Lymphoma | 0.000234 | 2.291 | 0.13 | 26 | 17304354 |
|  | Anaplastic Large Cell Lymphoma | 0.000595 | 2.521 | 0.17 | 26 | 17304354 |
|  |  |  |  |  |  |  |
| Ovarian | Ovarian Serous Adenocarcinoma | 0.00000242 | 11.828 | 0.05 | 53 | 19486012 |
|  | Ovarian Serous Cystadenocarcinoma | 0.00015 | 1.778 | 0.16 | 594 | TCGA |
|  |  |  |  |  |  |  |
| Pancreas | Pancreatic Carcinoma | 3.06E-05 | 1.533 | 0.07 | 52 | 19732725 |
|  |  |  |  |  |  |  |
| Prostate | Prostate Carcinoma | 2.15E-04 | -1.711 | 0.03 | 26 | 12154061 |
|  |  |  |  |  |  |  |
| sarcoma | Dedifferentiated Liposarcoma | 1.42E-14 | 1.568 | 0.01 | 55 | 20601955 |
|  | Pleomorphic Liposarcoma | 3.93E-10 | 1.972 | 0.02 | 32 | 20601955 |
|  | Myxoid/Round Cell Liposarcoma | 1.63E-11 | 1.507 | 0.02 | 29 | 20601955 |
|  | Myxofibrosarcoma | 2.33E-12 | 2.029 | 0.02 | 40 | 20601955 |
|  | Leiomyosarcoma | 6.15E-10 | 1.678 | 0.03 | 35 | 20601955 |
|  | Malignant Fibrous Histiocytoma | 0.000000315 | 5.338 | 0.01 | 24 | 15994966 |
|  | Leiomyosarcoma | 0.000159 | 3.679 | 0.04 | 21 | 15994966 |
|  | Synovial Sarcoma | 0.0000954 | 3.626 | 0.05 | 19 | 15994966 |
|  | Fibrosarcoma | 0.000492 | 4.573 | 0.06 | 22 | 15994966 |
| Others | Malignant Fibrous Histiocytoma | 0.000000315 | 5.338 | 0.01 | 24 | 15994966 |
|  | Skin Basal Cell Carcinoma | 0.000000369 | 2.885 | 0.01 | 19 | 18442402 |
|  | Skin Squamous Cell Carcinoma | 0.0000208 | 2.848 | 0.02 | 15 | 18442402 |
|  | Pleural Malignant Mesothelioma | 0.000131 | 2.324 | 0.06 | 49 | 15920167 |
|  | Vulvar Intraepithelial Neoplasia | 0.000356 | 2.772 | 0.06 | 19 | 17471573 |
|  | Testicular Yolk Sac Tumor | 0.000112 | -3.599 | 0.02 | 7 | 15994931 |
|  | Testicular Embryonal Carcinoma | 0.0000922 | -3.5 | 0.02 | 8 | 15994931 |
|  | Yolk Sac Tumor, NOS | 0.00000176 | -4.098 | 0.07 | 15 | 16424014 |
|  | Seminoma, NOS | 0.00000455 | -3.696 | 0.11 | 18 | 16424014 |
|  | Embryonal Carcinoma, NOS | 0.00000222 | -5.297 | 0.12 | 22 | 16424014 |
|  | Teratoma, NOS | 0.00000404 | -7.537 | 0.15 | 20 | 16424014 |
|  | Mixed Germ Cell Tumor, NOS | 0.00000367 | -4.656 | 0.15 | 47 | 16424014 |

**Table S3. CTHRC1 single-gene GO analysis in SangerBox database.**

| **GO ID** | **Nane Space** | **Name** | **RreDataBase** |
| --- | --- | --- | --- |
| GO:0016477 | biological_process | cell migration | GO_REF:0000107 |
| GO:0032092 | biological_process | positive regulation of protein binding | GO_REF:0000107 |
| GO:0033690 | biological_process | positive regulation of osteoblast proliferation | GO_REF:0000107 |
| GO:0043932 | biological_process | ossification involved in bone remodeling | GO_REF:0000107 |
| GO:0045669 | biological_process | positive regulation of osteoblast differentiation | GO_REF:0000107 |
| GO:0060071 | biological_process | Wnt signaling pathway, planar cell polarity pathway | GO_REF:0000107 |
| GO:0060122 | biological_process | inner ear receptor cell stereocilium organization | GO_REF:0000107 |
| GO:0090090 | biological_process | negative regulation of canonical Wnt signaling pathway | GO_REF:0000107 |
| GO:0090103 | biological_process | cochlea morphogenesis | GO_REF:0000107 |
| GO:0090177 | biological_process | establishment of planar polarity involved in neural tube closure | GO_REF:0000107 |
| GO:0005576 | cellular_component | extracellular region | PMID:27068509 |
| GO:0005581 | cellular_component | collagen trimer | GO_REF:0000037 |
| GO:0005615 | cellular_component | extracellular space | PMID:18467647 |
| GO:0005737 | cellular_component | cytoplasm | GO_REF:0000107 |
| GO:0062023 | cellular_component | collagen-containing extracellular matrix | PMID:28675934 |
| GO:0005109 | molecular_function | frizzled binding | GO_REF:0000107 |
| GO:0005201 | molecular_function | extracellular matrix structural constituent | PMID:28675934 |
| GO:0017147 | molecular_function | Wnt-protein binding | GO_REF:0000107 |
